# A Method for RNA Structure Prediction Shows Evidence for Structure in lncRNAs

**DOI:** 10.3389/fmolb.2018.00111

**Published:** 2018-12-03

**Authors:** Riccardo Delli Ponti, Alexandros Armaos, Stefanie Marti, Gian Gaetano Tartaglia

**Affiliations:** ^1^Centre for Genomic Regulation, Bioinformatics and Genomics Programme, The Barcelona Institute for Science and Technology, Barcelona, Spain; ^2^Universitat Pompeu Fabra, Barcelona, Spain; ^3^Institució Catalana de Recerca i Estudis Avançats, Barcelona, Spain; ^4^Department of Biology ‘Charles Darwin’, Sapienza University of Rome, Rome, Italy

**Keywords:** non-coding RNA, secondary structure, structural alignments, pair-wise comparisons, sequence-based predictions, RNA evolution

## Abstract

To compare the secondary structure profiles of RNA molecules we developed the *CROSSalign* method. *CROSSalign* is based on the combination of the Computational Recognition Of Secondary Structure (CROSS) algorithm to predict the RNA secondary structure profile at single-nucleotide resolution and the Dynamic Time Warping (DTW) method to align profiles of different lengths. We applied *CROSSalign* to investigate the structural conservation of long non-coding RNAs such as *XIST* and *HOTAIR* as well as ssRNA viruses including *HIV*. *CROSSalign* performs pair-wise comparisons and is able to find homologs between thousands of matches identifying the exact regions of similarity between profiles of different lengths. In a pool of sequences with the same secondary structure *CROSSalign* accurately recognizes repeat A of *XIST* and domain D2 of *HOTAIR* and outperforms other methods based on covariance modeling. The algorithm is freely available at the webpage http://service.tartaglialab.com//new_submission/crossalign.

## Introduction

Sequence similarity is often considered the key feature to investigate evolutionary conservation of coding transcripts (Kent, 2002). Yet, knowledge of secondary structure provides important insights into the biological function of RNAs by allowing the study of physical properties, such as for instance molecular interactions (Bellucci et al., 2011). In most cases, information about the RNA folding complements sequence analysis (Wan et al., 2014) and is useful to understand their mechanisms of action. MicroRNA precursors, for example, are processed by DGCR8 only if properly folded in hairpin loop structures (Ha and Kim, 2014). Similarly, the architecture of ribosomal RNAs evolves in a self-contained way through conservation of stem loops present in ancient species (Bokov and Steinberg, 2009, p. 200; Petrov et al., 2015), indicating distinct requirements for structural elements.

Long non-coding RNAs (lncRNAs) are regarded as a mystery in terms of sequence and structural conservation (Ulitsky, 2016). The vast majority of lncRNAs seems to evolve under little or no selective constraints, undergo almost no purifying selection, show poor expression, and do not have often easily identifiable orthologs (Diederichs, 2014; Ulitsky, 2016). Indeed, the average sequence homology of evolutionarily conserved lncRNAs between human and mouse is 20% and drops to 5% between human and fish (Ulitsky, 2016). Thus, primary structure does not provide relevant information to study lncRNA conservation and secondary structure could be used for better characterization. Similarly to lncRNAs, the transcriptomes of single-stranded RNA (ssRNA) viruses retain their fold even if sequences mutate rapidly (Chursov et al., 2013), which indicates that secondary structure investigation could be key to reveal evolutionary properties.

To study the structural conservation of two RNA molecules, we developed the *CROSSalign* method. *CROSSalign*, available at our webpages http://service.tartaglialab.com//new_submission/crossalign, is based on the combination of two methods: (1) Computational Recognition Of Secondary Structure (CROSS), which is an algorithm trained on experimental data to predict RNA secondary structure profiles without sequence length restrictions and at single-nucleotide resolution (Delli Ponti et al., 2017); (2) the Dynamic Time Warping (DTW) algorithm to assess the similarity of two profiles of different lengths (Giorgino, 2009). DTW flexibility allows managing profiles of different lengths without having to sacrifice computational time. The comparisons of structural profiles will lead to a broad applicability of our methodology. Indeed, profiles have already started to be employed to assess structural similarities of large molecules such as ribosomal RNAs (Lavender et al., 2015).

We applied *CROSSalign* on lncRNAs of different species, comparing our results with those of covariation models based on multiple-alignments. *CROSSalign* is able to find structural homologs among millions of possible matches identifying structural domains with great accuracy. The algorithm is able to recognize RNAs with low-sequence/high-structure similarity, which allows to better identify the physico-chemical properties of RNA molecules.

## Results

To test the performances and functionality of CROSS combined with DTW (Supplementary Figures 1, 2), we selected a dataset of 22 structures for which crystallographic (exact base pairing between nucleotides) and Selective 2'-hydroxyl acylation analyzed by primer extension (SHAPE; chemical probing of flexible regions used to assess whether a nucleotide is in double- or single-stranded state) data are available (Lorenz et al., 2016). Using DTW, we calculated the distances between all possible pairs in the dataset considering crystallographic (dots and parentheses were transformed into binary code) data as well as (1) SHAPE profiles (Area Under the ROC Curve AUC of 0.76, Positive Predictive Value PPV of 0.76 when compared to crystallographic data) and (2) CROSS profiles (AUC 0.72 and PPV 0.74 when compared to crystallographic data, see http://service.tartaglialab.com/static_files/algorithms/cross/documentation.html#5).

**Figure 1:**
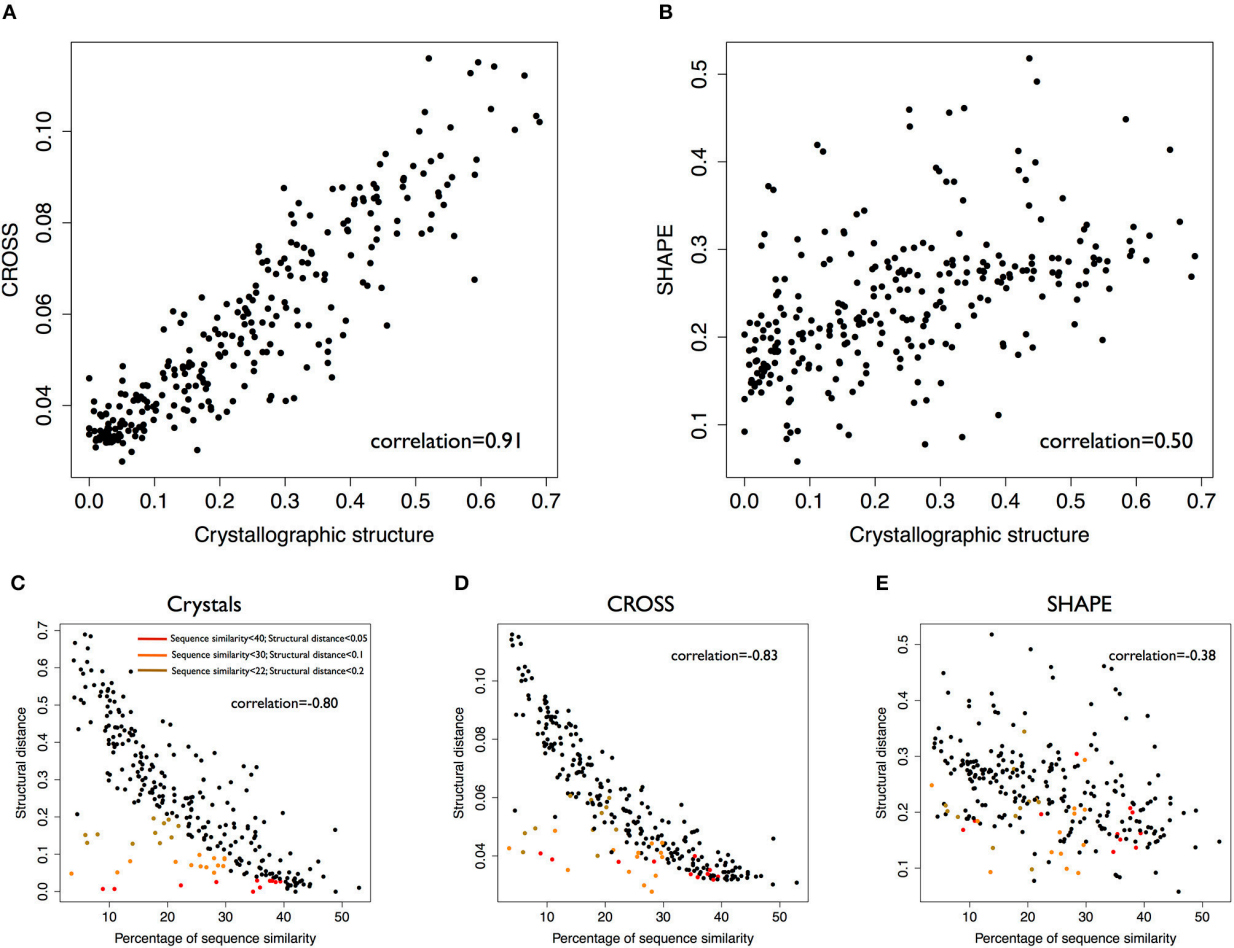
Validation of the CROSSalign method. **(A)** Correlation between distances computed with CROSS and crystallographic profiles on 22 structures (*standard-DTW*). **(B)** Correlation between distances computed with SHAPE and crystallographic data on the same data (*standard-DTW*). **(C)** Correlation between structural distances (crystallographic profiles) and sequence similarity. Clusters of similar structures and different sequences (sequence similarity < 40%) are highlighted in brown (structural score < 0.2), orange (structural score < 0.1), and (red structural score < 0.05). **(D)** Correlation between structural distances (*CROSSalign* distances) distances and sequence similarity. The clusters previously identified for crystallographic data are shown in the plot. **(E)** Correlation between structural distances (SHAPE) and sequence similarity. In this case the clusters are disrupted.

Crystallographic data show higher correlations with CROSS profiles (Pearson's correlation of 0.91) than SHAPE profiles (Figures 1A,B, correlation of 0.50). In this analysis, CROSS shows better performances than algorithms such as *RNAstructure* (Mathews et al., 1999; Reuter and Mathews, 2010) and *RNAfold* (Lorenz et al., 2016) (respective correlations 0.71 and 0.47 with crystals; Supplementary Figures 3, 4).

Sequence similarity analysis (computed with EMBOSS; Material and Methods) shows comparable correlations with distances calculated with either CROSS or crystallographic profiles (respectively: 0.80, 0.83, 0.38 with crystallographic, CROSS and SHAPE data). While CROSS and crystallographic profiles identify specific clusters of RNA molecules with low sequence identity and high structural similarity (colored in red, orange and green according to difference confidence thresholds; Figures 1C–E), SHAPE data cannot be used to recognize these structures.

To further test the usefulness of *CROSSalign*, we compared its output with that of *CMfinder* (Yao et al., 2006). *CMfinder* is a method to compute multiple sequence alignments that exploits structural motifs for ranking (section Materials and Methods, *Comparisons with CMfinder*). We analyzed the largest multiple sequence alignments reported in the *CMfinder* test set (cobalamin, intron gp II, s box, lysine and histone 3) and used the minimal *CROSSalign* distance to assign the closest match to each transcript. This step is needed for the analysis, since *CROSSalign* performs pairwise comparisons, while *CMfinder* does multiple alignments. Selecting equal-size groups (lowest and highest *CMfinder* scores), we measured *CROSSalign* performances on *CMfinder* rankings, achieving an AUC of 0.80 (Supplementary Figure 5A). We note that *CROSSalign* has particularly strong performances on the largest dataset: cobalamin (71 sequences of 216 nt; AUC of 0.95; Supplementary Figure 5B).

### Ribosomal RNAs

Ribosomal RNAs are considered one of the most ancient, structured and conserved classes of RNA molecules (Bokov and Steinberg, 2009). The first step to validate *CROSSalign* was to search for structural similarities between the *Small SubUnit* (SSU) of the rRNA of different bacteria (*Pseudomonas aeruginosa, Escherichia coli, Bacillus subtilis, Deinococcus radiodurans*). All the rRNAs show significant structural similarity (*p*-value < 10^−5^) with a low *CROSSalign* distance (~0.08; Table 1A). By contrast, shuffling one of the two sequences in *CROSSalign* predictions results in non-significant scoring (*p*-values of 0.10 or higher; Table 1A), indicating the importance of the sequence context in our calculations.

**Table 1:** Conservation of ribosomal structures.

	***B. subtilis***	***D. radiodurans***	***E.coli***	***P. aeruginosa***	***E. coli* (shuffled)**
**A**					
*B. subtilis*	**0**	**0.086** (*p*-value < 10^−5^)	**0.088** (*p*-value < 10^−5^)	**0.082** (*p*-value < 10^−5^)	0.098 (*p*-value = 0.19)
*D. radiodurans*	0.10 (*p*-value = 0.35)	**0**	**0.085** (*p*-value < 10^−5^)	**0.087** (*p*-value < 10^−5^)	0.10 (*p*-value = 0.35)
*E. coli*	0.098 (*p*-value = 0.19)	0.10 (*p*-value = 0.35)	**0**	**0.066** (*p*-value < 10^−5^)	0.098 (*p*-value = 0.19)
*P. aeruginosa*	0.097 (*p*-value = 0.13)	0.10 (*p*-value = 0.35)	0.098 (*p*-value = 0.19)	**0**	0.098 (*p*-value = 0.19)
	***E. Coli***		***E. coli*** **(shuffled)**		***H. sapiens (mRNA)***
**B**					
*H. sapiens*	**0.079** (*p*-value < 10^−5^)		0.097 (*p*-value = 0.13)		0.096 (*p*-value = 0.10)
*P. furiosus*	**0.077** (*p*-value < 10^−5^)		0.096 (*p*-value = 0.10)		0.096 (*p*-value = 0.10)
*S. cerevisiae*	**0.079** (*p*-value < 10^−5^)		0.100 (*p*-value = 0.35)		0.095 (*p*-value = 0.05)

***(A)** Above the diagonal: CROSSalign distances and p-values of bacterial SSUs; below the diagonal: structural distances and p-values upon shuffling one of the two bacterial sequences (Genbank/NCBI: J01859.1, NR_026078, NR_074411.1, bacillus and NR_102783.2). **(B)** Structural distance and p-values of SSUs against E. coli (Genbank/NCBI: M10098.1, NR_074375.1, and NR_132222.1). The shuffled sequence of Escherichia coli is used as a negative control. A coding human mRNA randomly selected from ENSEMBL with the same length of E. coli ribosome (ENSG00000002933) is used as an additional control to highlight the exclusive structural similarities between the different SSUs. Significant p-values are reported in bold*.

*Pseudomonas aeruginosa* and *E. coli* are the most similar SSUs (*CROSSalign* distance of 0.06; *p*-value < 10^−5^). As the SSU of the rRNAs is thought to have evolved in a self-contained structure where the secondary structure of the ancient species is contained in the recent ones (Petrov et al., 2015), we searched the complete SSU of *E. coli* in the SSU of other species such as *Pyrococcus furiosus, Saccharomyces cerevisiae* and *Homo sapiens*. The results show a strong and significant structural similarity, in agreement with the theory of self-contained evolution (Table 1B). By contrast, comparison with randomized *E. coli* SSU or *H. sapiens* mRNA of the same length results in non-significant scores (*p*-values of 0.10 or higher Table 1B).

### XIST

**Figure 2:**
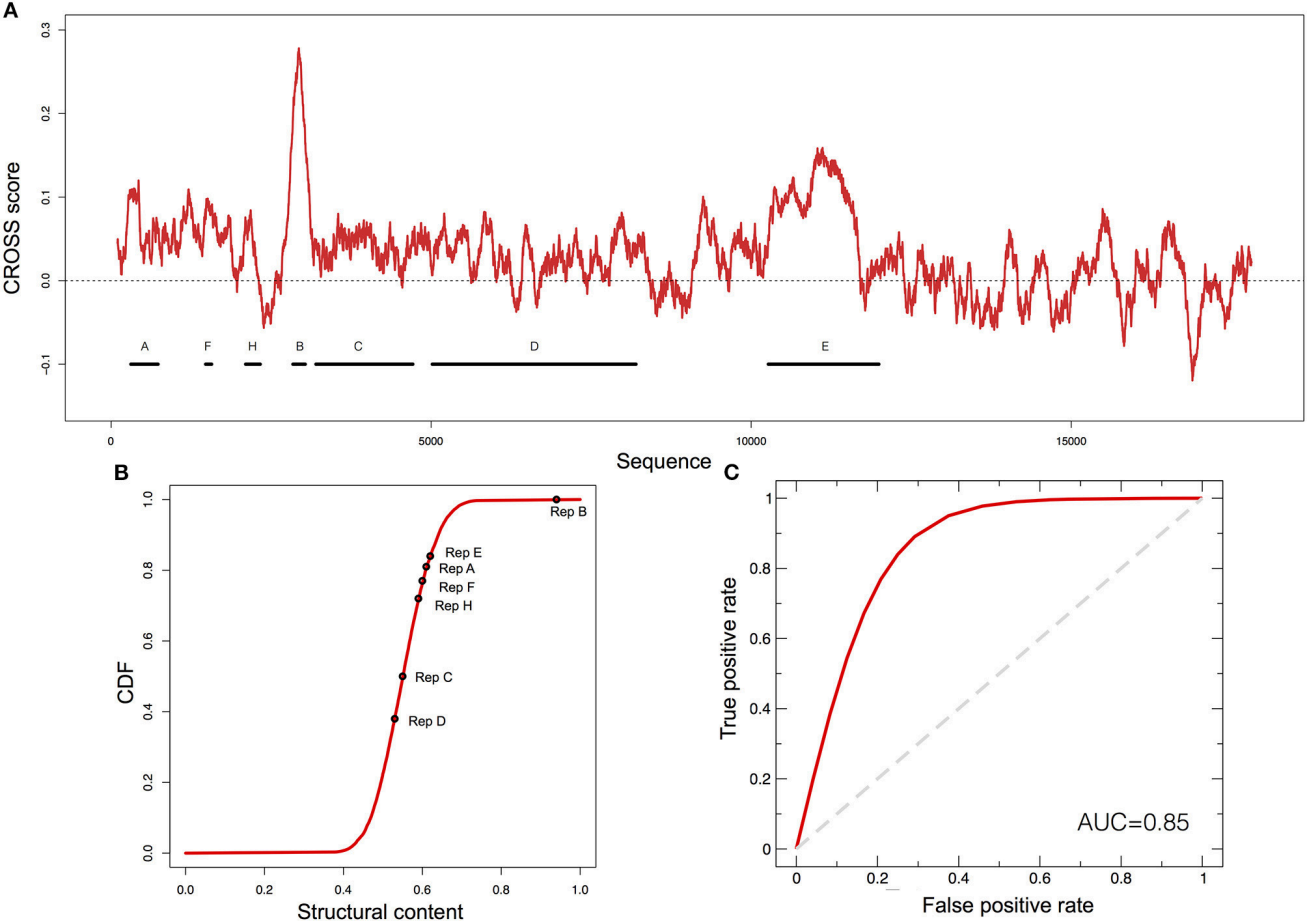
Predictions of XIST structure. **(A)** Secondary structure profile of murine *XIST* obtained using CROSS. Positive regions are to be considered double-stranded, negative regions single-stranded. **(B)** Cumulative distribution function (CDF) for the structural content of all the human lincRNAs predicted by CROSS. The structural contents of the Rep domains of *XIST* are reported on the curve. **(C)** ROC curve of *CROSSalign* to identify reverse engineered sequences with the same structure as RepA and a different sequence.

*XIST* is a lncRNA characterized by several repetitive domains showing different structural properties (Figures 2A,B) (Pintacuda et al., 2017). The 5' conserved region, named RepA (or A-repeat), is indispensable for gene-silencing and has been shown to be highly conserved in mammals. In mouse it consists of 7.5 copies (8.5 in humans) of 26-mers separated by U-rich linkers (Figure 2A) (Brockdorff, 2002). In 2015 and 2017, two reports on the *XIST* A-repeat structure were published (Fang et al., 2015; Liu et al., 2017), both making use of experimental techniques to infer *XIST* structure. The RepA structures obtained are similar with strikingly identical stem-loop structures, both emerging from larger RNA bulges of repeats 3, 5, and 6 [for a comparison with CROSS predictions, see our previous manuscript (Delli Ponti et al., 2017)].

To evaluate the ability of *CROSSalign* to recognize the structural content in RepA, we used *RNAinverse* from Vienna suite (Lorenz et al., 2016) to generate 50 different sequences (Supplementary Table 1) with the same structure as RepA (Materials and Methods; *Reverse engineering: from structure to sequence*). The sequences were then divided into a reference (25 transcripts) and a positive (25 transcripts) set, and we built a list of negative cases by shuffling 25 times the original RepA. Both the positive and negative sets have poor sequence similarity (<35% computed with EMBOSS; see Materials and Methods). We used *CROSSalign* to compute all scores for the positive and negative set against the corresponding reference set and used the minimal distance to assign the closest match to each transcript. The strong performances obtained highlight the ability of *CROSSalign* to identify structural similarities regardless of the sequence similarity (AUC of 0.85; Figure 2C).

As a further test of the usefulness of *CROSSalign* we compared the above results with those obtained using *CMsearch* from the Infernal-1.1.2 package, an algorithm based on a covariance model approach (Nawrocki and Eddy, 2013). In this case, *CMsearch* is not able to identify any match between either the positive or negative list and the reference sets (AUC of 0.5; section Materials and Methods; *Comparisons with CMsearch*). Indeed, the sequence similarity of the positive set to the RepA is negligible (Supplementary Figure 6), which affects *CMsearch* performances. The results indicate that *CROSSalign* is able to recognize structural similarities between non-similar sequences, outperforming covariance-based approaches.

After this first validation step, we used *CROSSalign* to study structural similarities of *XIST* domain RepA in 10 different species (Rivas et al., 2017). Our analysis reveals that primates cluster close to human (Supplementary Figure 7A) while other species are more distant (Supplementary Figures 7A, 8). By contrast, calculating sequence similarity with respect to human *XIST* (computed with EMBOSS; see section Materials and Methods), we could not identify a specific cluster for primates (Supplementary Figure 7B). Thus, our results indicate that secondary structure shows a higher degree of conservation than sequence.

**Figure 3:**
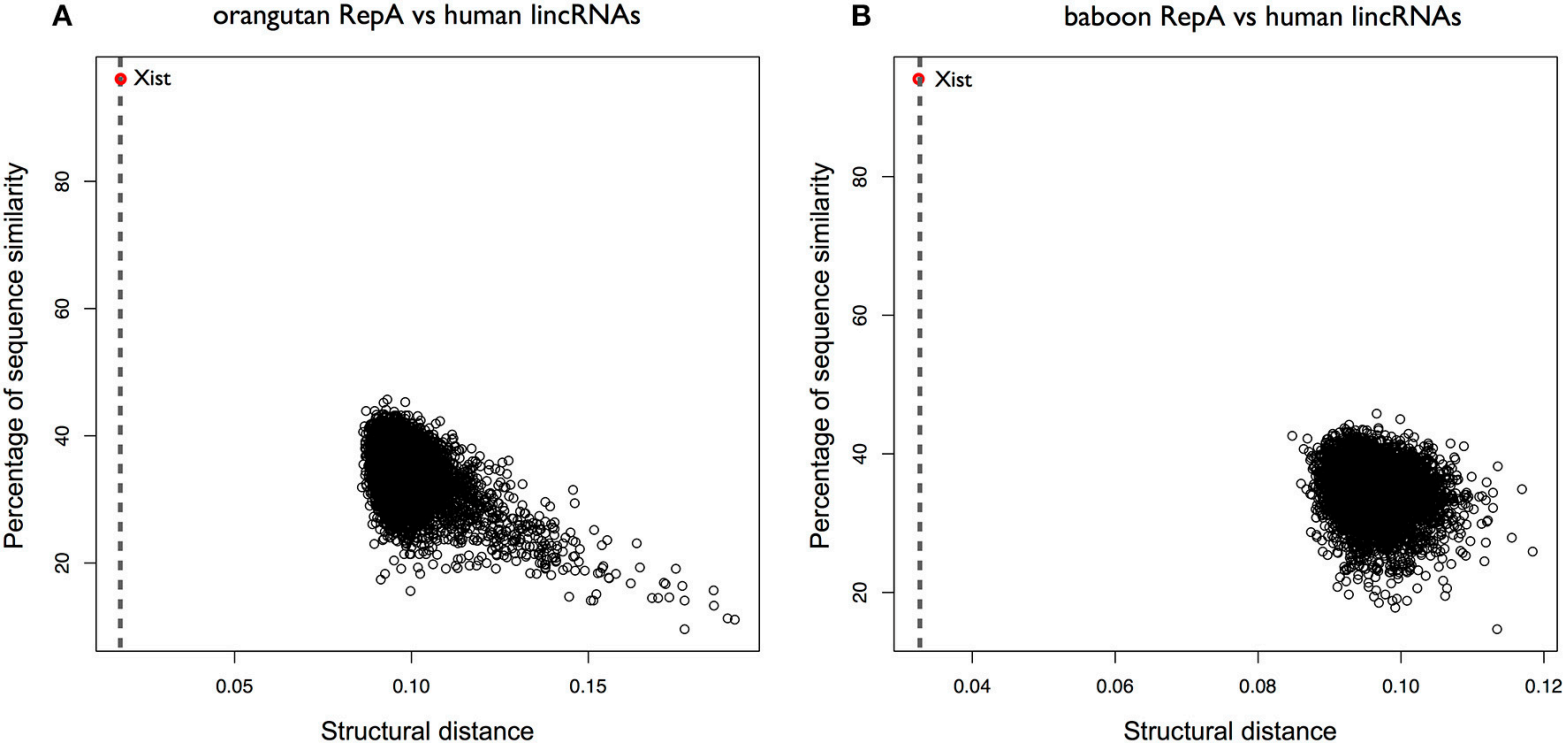
Structural conservation of XIST RepA within primates. **(A)** *CROSSalign* distances of Orangutan RepA are computed against all human lincRNAs. The distance is calculated using *OBE-DTW* and plotted against sequence similarity. Orangutan RepA is identified as the best match (colored in red). **(B)** *CROSSalign* distances of baboon RepA against all the lincRNAs of human. Baboon RepA is identified as the best match (colored in red).

We then selected RepA of orangutan and searched for structural similarities within all human intergenic lncRNAs (lincRNAs 8176 sequences; ENSEMBLE 84). *XIST* was ranked as the best significant match in the pool (*CROSSalign* distance 0.01; *p*-value < 10^−6^) and RepA was correctly identified (predicted coordinates: 328–764; 95% overlap with the query region; Figure 3A; Supplementary Table 2). Similar results were observed for baboon RepA (best result: 0.032; 86% overlap with the query region) and lemur RepA (best result: 0.075; *p*-value; 97% overlap with the query region), suggesting a strong structural conservation within primates (Figure 3B). By contrast, human and mouse RepA showed a larger distance in terms of both structural and sequence similarity, which is in agreement with previous studies on lncRNA conservation (Breschi et al., 2017).

**Figure 4:**
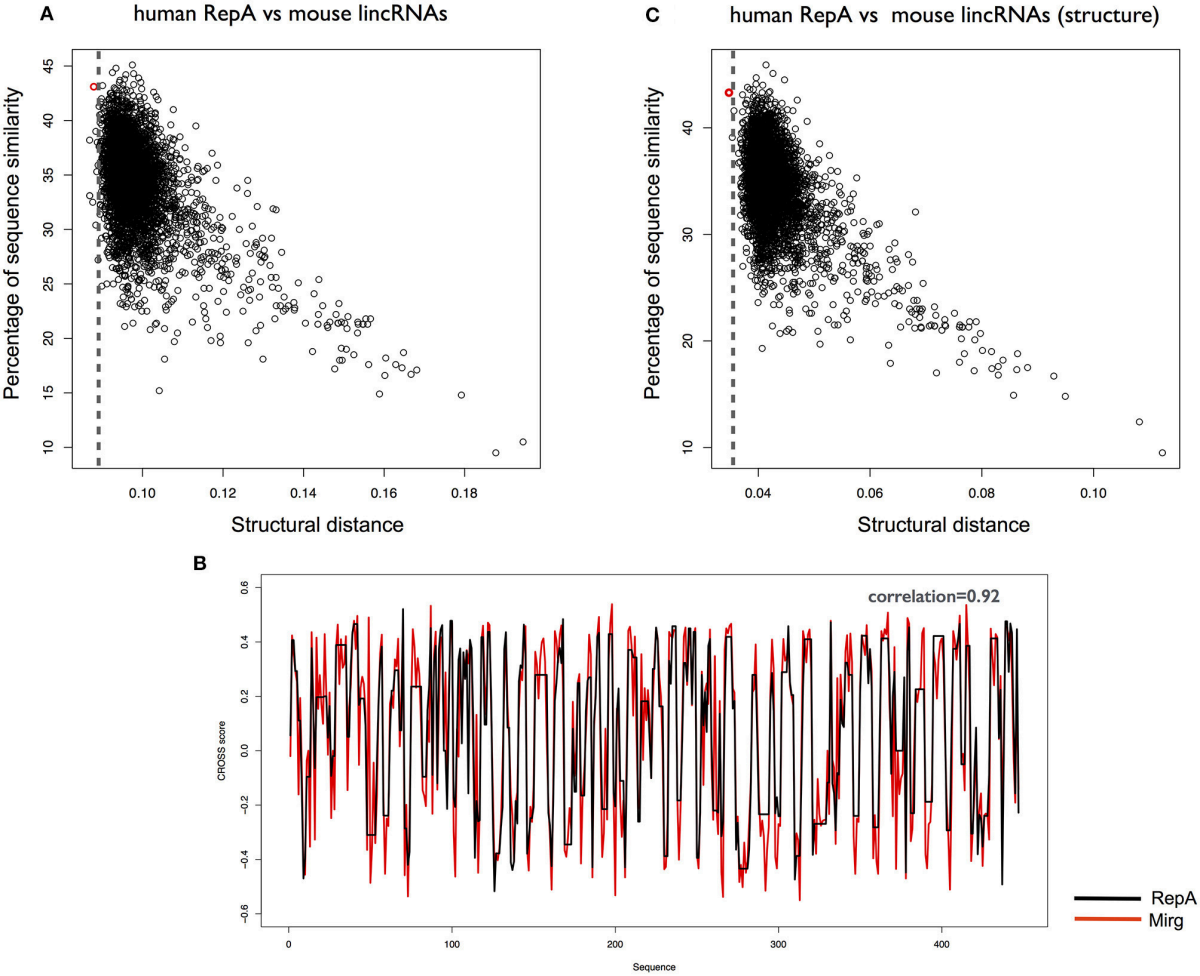
Structural similarities between human and mouse *XIST* RepA. **(A)** Structural similarities of human RepA against all mouse lincRNAs. The distance was calculated using CROSSalign (*OBE-DTW*) and plotted against sequence similarity. Human RepA is not the best match (5th best hit; colored in red). **(B)** Structural similarities of human RepA against all mouse lincRNAs using double-stranded nucleotides (nucleotide with CROSS score < 0 are set to 0). Human RepA is identified as the best match (colored in red), which highlights the importance of the structural content for the regulatory domains of the lncRNAs. **(C)** Secondary structure profile of human RepA, obtained as optimal path with *OBE-DTW*, compared with the best match in mouse lincRNAs (*Mirg*; ENSMUSG00000097391). The two secondary structure profiles show a strong a correlation (0.92).

We used *CROSSalign* to search for the human RepA within all mouse lncRNAs and identified *XIST* as the 5th best hit (*CROSSalign* distance 0.085; *p*-value < 10^−6^; Figure 4A). In this case, the position of RepA was not correctly assigned (coordinates: 10306–10698; 0% overlap) but the best match falls into the regulatory region of exon 7, and the structural relation between RepA and exon 7 has been reported (Yamada et al., 2015). Importantly, the correct coordinates of human RepA within mouse *XIST* rank second in our analysis (*CROSSalign* distance 0.086; *p*-value < 10^−6^), while the best match is a miRNA-containing the gene *Mirg* (ENSMUSG00000097391) and the two secondary structure profiles show a strong correlation of 0.92 (Figure 4B). Interestingly, even if little information is available on *Mirg*, the transcript is prevalently expressed in the embryo (Schmitt et al., 2014). This result unveils a previously unreported relationship between *XIST* and *Mirg*, in which structural and functional homologies can be linked. Intriguingly, also the second best result, *Rian* (ENSMUSG00000097451), is expressed in embryo, while no information is available on the third and fourth hits (ENSMUSG00000107391 and ENSMUSG00000085312). We note that the five matches here are not listed in the top 20 hits obtained by analysis of sequence similarity (<34%).

Our results suggest that the secondary structure of RepA is conserved among primates, and diverges between human and mouse. However, analyzing the information contained in structured nucleotides (i.e., nucleotides with CROSS score < 0 are set to 0) we could identify *XIST* as the best match of human RepA within all mouse lncRNAs (*CROSSalign* distance 0.034; *p*-value < 10^−6^; Figure 4C). This result indicates that double-stranded regions are more conserved than single-stranded regions. By sequence identity, *XIST* ranks as the 14th hit of human RepA in all mouse lncRNAs, which indicates that methods based on sequence comparison show a significantly lower ability to identify structural homologs.

### HOTAIR

**Figure 5:**
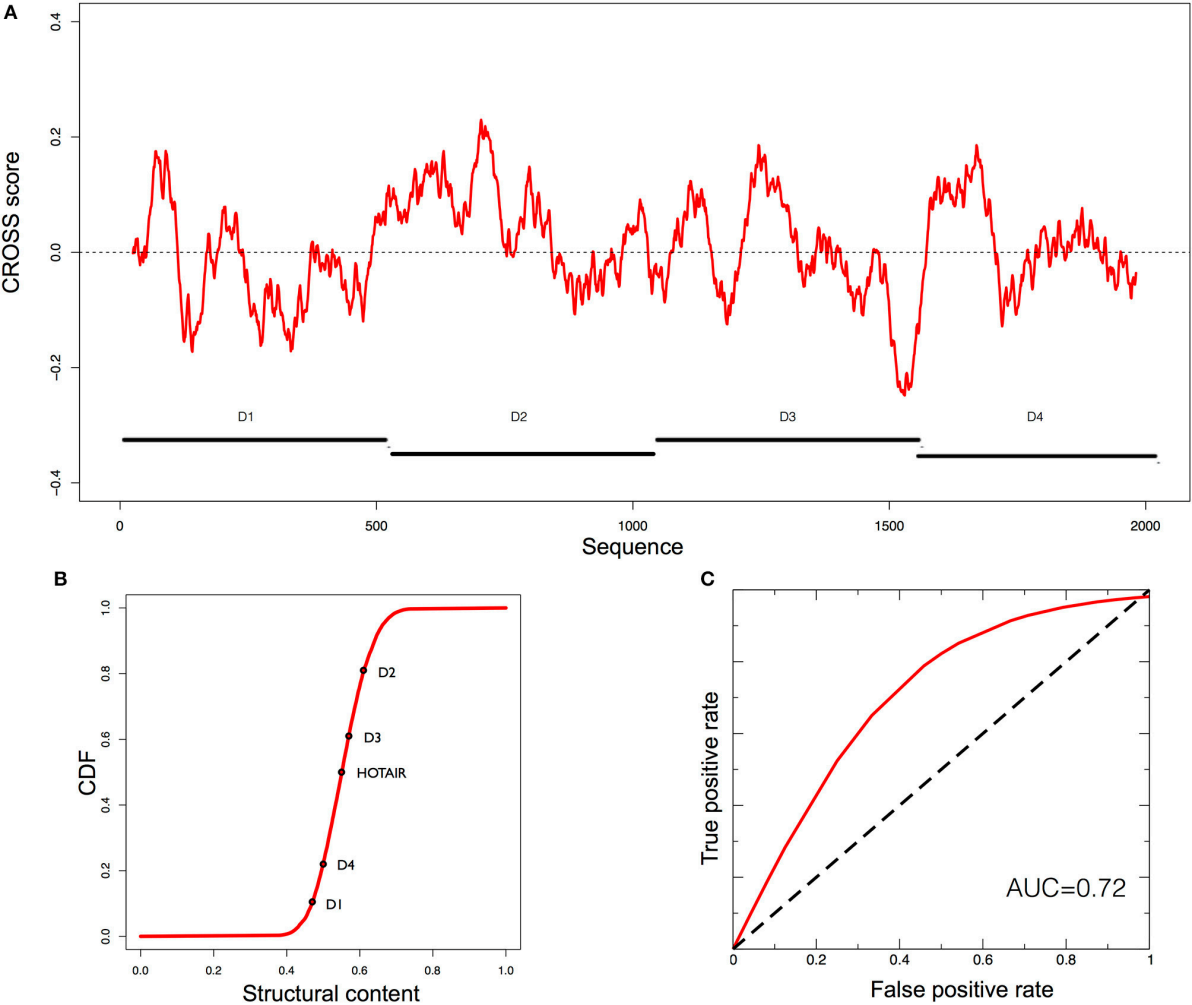
Predictions of HOTAIR structure. **(A)** Secondary structure profile of the complete murine *HOTAIR* obtained using CROSS. Positive regions are to be considered double-stranded, negative regions single-stranded. **(B)** Cumulative distribution function (CDF) for the structural content of all the human lincRNAs predicted by CROSS. The structural contents of the D domains of *HOTAIR* are reported on the curve. **(C)** ROC curve of *CROSSalign* to identify reverse engineered sequences with the same structure as D2 and a different sequence.

*HOTAIR* shows a complex secondary structure, divided into four domains (D1, 2, 3, 4; Figures 5A,B) (Somarowthu et al., 2015). Experimentally it has been determined that more than 50% of the nucleotides are involved in base pairing (CROSS achieves an AUC > 0.80 in predicting its SHAPE profile; Supplementary Figures 9A,B) (Somarowthu et al., 2015). The region D2 is highly structured and consists of 15 helices, 11 terminal loops, and 4 junctions (three 5-way junctions and one 3-way junction) (Somarowthu et al., 2015).

The D2 domain of *HOTAIR* is predicted by *CROSS* to be the most structured (Figure 5B). We used the same reverse engineering process as for RepA to generate 50 different sequences (Supplementary Table 1) with the same secondary structure as D2 (section Materials and Methods; *Reverse engineering: from structure to sequence*). The sequences generated by *RNAinverse* showed a similarity that is higher (average identity of 40% calculated with EMBOSS; see section Materials and Methods) than in the RepA case (Supplementary Figure 6), Even in this case, *CROSSalign* reports good performances (AUC of 0.72; Figure 5C) that are better than those of the covariance-based approach *CMsearch*, which depends on sequence similarity (AUC of 0.70; Supplementary Figure 10; section Materials and Methods; *Comparisons with CMsearch*) (Nawrocki and Eddy, 2013).

**Figure 6:**
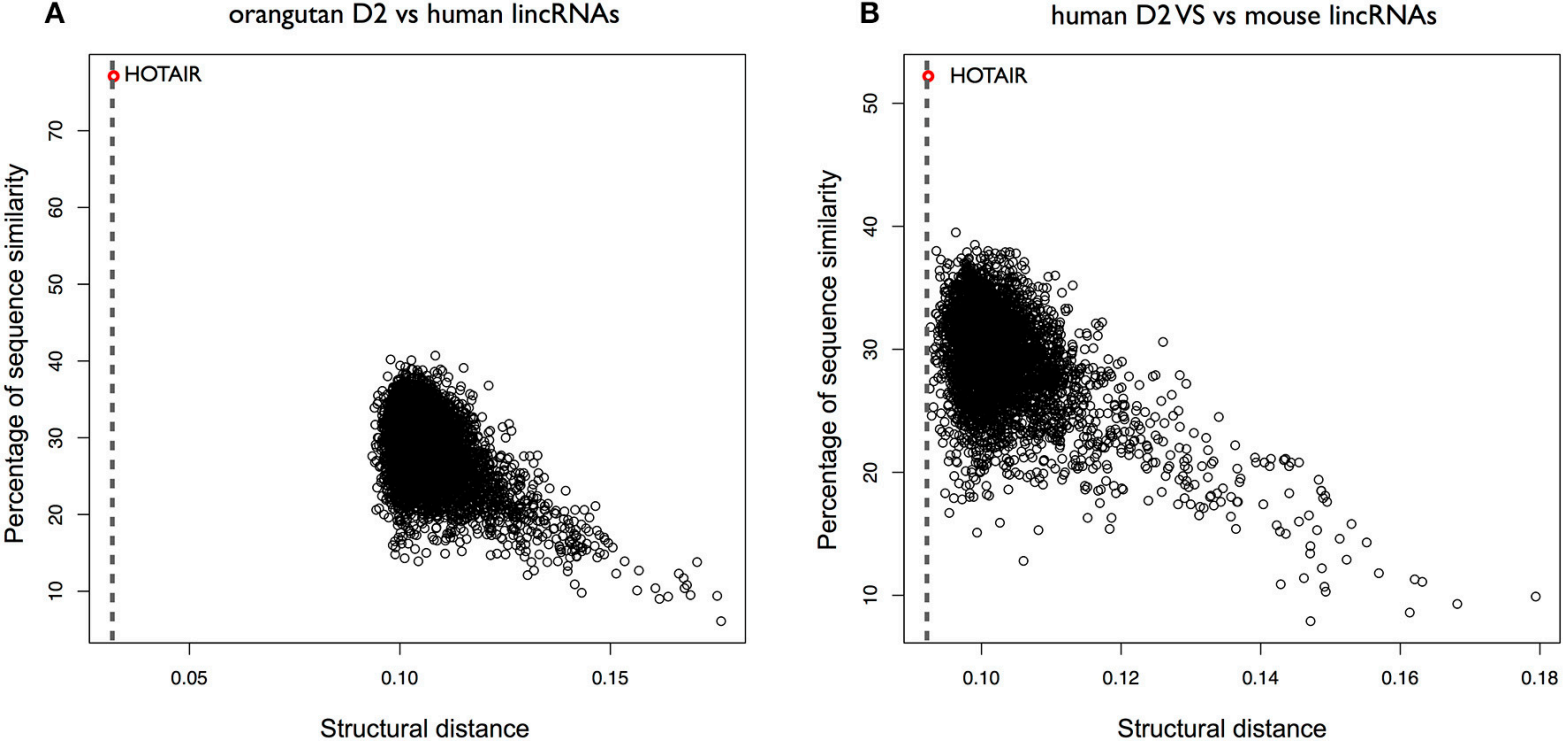
Structural conservation of HOTAIR D2 in different species. **(A)** Structural similarities of orangutan D2 against all human lincRNAs. The distance was obtained using *OBE-DTW* and plotted against with the sequence similarity. The D2 of orangutan is identified as the best match (colored in red). **(B)** Structural similarities of human D2 against all mouse lincRNAs. Human D2 is identified as the best match (colored in red).

We then selected the D2 domain of *HOTAIR* to measure its conservation in 10 species (Rivas et al., 2017) using *CROSSalign* (Supplementary Figure 11A). As for *XIST*, the distance analysis indicates that primates cluster close to human, and other species are more distant (Supplementary Figure 11B). Orangutan D2 was then searched for within all human lncRNAs, and *HOTAIR* was identified as the best match (*CROSSalign* distance 0.032; *p*-value < 10^−6^) with overlapping coordinates (nucleotides: 666–1191; 78% overlap with the query region; Figure 6A). Searching for mouse D2 within all human lncRNAs, *HOTAIR* was found as the best (0.092; *p*-value < 10^−4^) and matching position (nucleotides: 284–788; 57% overlap; Figure 6B). These results suggest that D2 secondary structure is not only conserved in primates but also in mouse.

**Figure 7:**
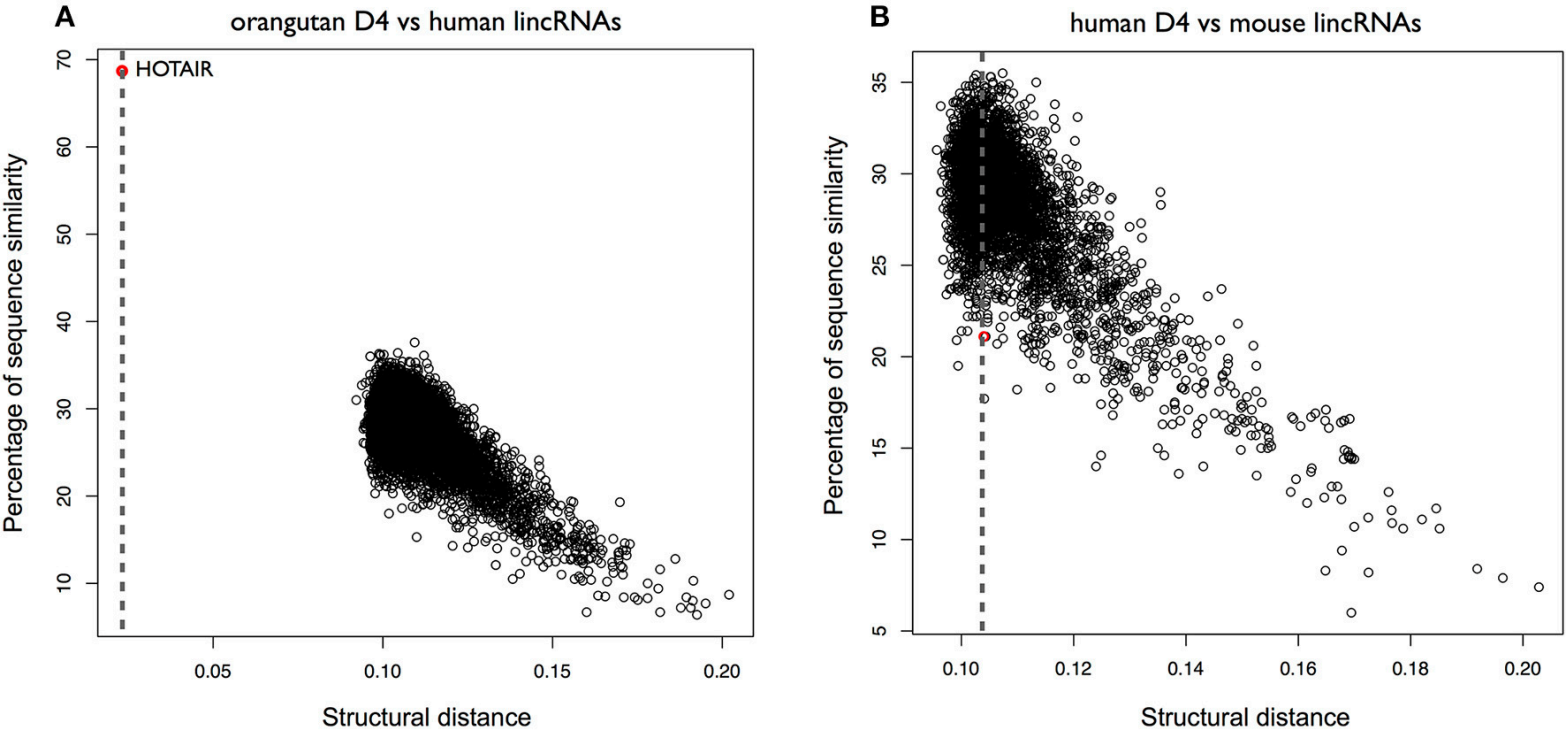
Structural conservation of HOTAIR D4 in different species. **(A)** Structural similarities of Orangutan D4 against all human lincRNAs. The distance was obtained using *OBE-DTW* and plotted against sequence similarity. Human D4 is identified as the best match (colored in red). **(B)** Structural similarities of human against all mouse lincRNAs. Human D4 is not identified as the best match (1849th best hit; colored in red).

To further investigate the secondary structure of *HOTAIR*, we studied the structural conservation of the D4 domain (Supplementary Figure 12). As opposed to D2, D4 is predicted by CROSS to be poorly structured (Figure 5B). Searching for orangutan D4 within all human lncRNAs yields HOTAIR as the best match (*CROSSalign* distance 0.023; *p*-value < 10^−6^) and the reported sequence position shows a sizeable overlap with the D4 domain in human HOTAIR (predicted coordinates: 1650–2291; overlap of 79%; Figure 7A). By contrast, when searching for mouse D4 within all human lncRNAs, HOTAIR shows poor ranking (1849th; *CROSSalign* distance 0.104; *p*-value = 0.061), which indicates little structural homology between human and mouse (Figure 7B; Supplementary Figure 10).

### HIV

HIV is one of the most studied ssRNA viruses with a complex secondary structure (Watts et al., 2009) that is accurately predicted by CROSS (Delli Ponti et al., 2017) (see also http://service.tartaglialab.com/static_files/algorithms/cross/documentation.html#4). As other organisms, HIV and ssRNA also contain non-coding regions (Wang et al., 2017).

We divided HIV into 10 non-overlapping regions of ~1,000 nucleotides and searched each of them against a database of ssRNA viruses having as host human (292 cases, downloaded from NCBI; Supplementary Table 3) to identify structurally similar domains. We found that coronavirus HKU and Simian-Human immunodeficiency SIV have the most significant matches with HIV (*CROSSalign* distance 0.078, *p*-value < 10^−6^ for SIV; *CROSSalign* distance 0.093, *p*-value < 10^−4^ for HKU). This finding is particularly relevant since SIV and HIV share many similarities in terms of pathogenicity and evolution (Sharp and Hahn, 2011). Indeed, previous studies already reported a similarity in terms of secondary structure between HIV and SIV that is not explained by sequence similarity (Rizvi and Panganiban, 1993).

**Figure 8:**
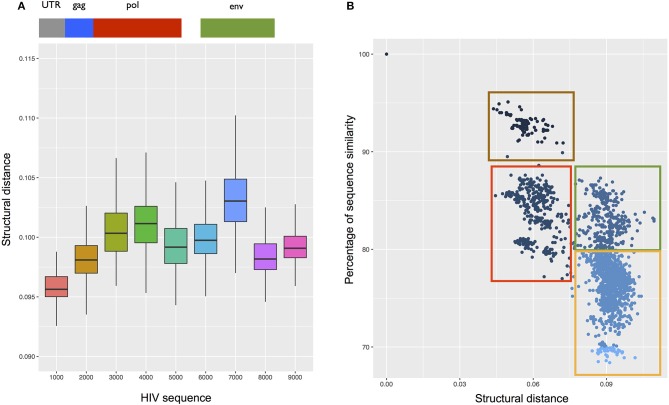
Structural analysis of the HIV transcriptome. **(A)** Structural conservation of HIV genome (divided into 10 not overlapping regions) compared with the complete genome of 292 ssRNA viruses. The region spanning the first 1000 nt (including 5′ UTR) is the most conserved among all the viruses. **(B)** *CROSSalign* distances of the complete HIV genome against the complete genomes of 4884 HIV strains. Using analysis of primary and secondary structures, we identified four main clusters (red, green, brown, and yellow). Red and green boxes indicate strains whose structural difference cannot be identified through sequence analysis, while brown and red boxes as well as green and yellow boxes identify strains with similar structures and different sequences.

In addition, we found that the HIV 5′ region is structurally similar to a strain of Ebola virus (Tai Forest; Supplementary Table 3). In agreement with this observation, previous studies indicate that HIV and Ebola have the same mechanisms of egress, taking contact with the cellular protein Tsg101 (Martin-Serrano et al., 2001). Moreover, HIV 5′ is the most conserved region in all ssRNA viruses (Figure 8A). This result indicates that the secondary structure of this region is not only necessary for HIV encapsidation (Lu et al., 2011), but is also essential for the activity of other viruses.

We also compared *CROSSalign* distances and sequence similarities of all HIV strains (4804; see section Materials and Methods). We found two clusters (brown and red; Figure 8B) that are similar in terms of structure (~0.06 *CROSSalign* distance; *p*-value < 10^−6^) and sequence (80–95% sequence similarity). Other clusters (red and green; Figure 8B) showed significant distance in structure (from 0.06 to 0.09 of *CROSSalign* distance; *p*-value < 10^−6^) that is not identifiable by sequence similarity (~85% sequence similarity). This result suggests that HIV could have evolved maintaining a similar sequence but different structures, as previously reported in literature (Rizvi and Panganiban, 1993).

## Discussion

We developed the *CROSSalign* method based on the combination of the CROSS algorithm to predict the RNA secondary structure at single-nucleotide resolution (Delli Ponti et al., 2017) and the DTW algorithm to align profiles of different lengths (Giorgino, 2009). DTW has been previously applied in different fields, especially pattern recognition and data mining (Keogh and Pazzani, 2000; Rath and Manmatha, 2003), but has never been used to investigate structural alignments of RNA molecules. Since CROSS has no sequence length restrictions and shows strong performances on both coding and non-coding RNAs (Delli Ponti et al., 2017) the combination with DTW allows very accurate comparisons of structural profiles. On our test set, thermodynamic approaches such as *RNAstructure* (Reuter and Mathews, 2010) and *RNAfold* (Lorenz et al., 2016) showed appreciable but yet lower performances than CROSS. Moreover, their restrictions on sequence length (Lu et al., 2009; Hajiaghayi et al., 2012) limit the applicability to large domains (Cirillo et al., 2017).

We applied *CROSSalign* to investigate the structural conservation of lncRNAs in different species and the complete genomes of ssRNA viruses. We found that the algorithm is able to find structural homologs between thousands of matches and correctly identifies the regions of similarity between profiles of different lengths. The results of our analysis reveal a structural conservation between known lncRNA domains including *XIST* RepA (best hit out of 8,176 cases; 95% overlap with the query region; Figure 3A) and *HOTAIR* D2 (best hit out of 8,176 cases; 78% overlap with the query region; Figure 6A), but also identify structural similarities between regulatory regions of HIV and other ssRNA viruses (Figure 8), opening new questions regarding similar mechanisms mediated by the secondary structure.

RepA and D2 profiles were accurately recognized in a pool of RNAs designed to have the same structure but different sequences. Indeed, the reverse engineering analysis performed with *RNAinverse* shows that *CROSSalign* accurately recognizes (AUC of 0.85; Figure 2C) highly dissimilar sequences (< 35%) encoding for the same RepA structure. On the same datasets, multiple sequence alignments performed with *CMsearch* show lower performances. These results mirror our findings for crystallographic data indicating that *CROSSalign* is able to identify clusters of low-sequence / high-structure similarity (Figure 1D). Indeed, the algorithm proves to be very useful for the identification of structural similarities that are not captured through multiple-sequence alignments.

As shown in the case of mouse RepA, aligning the structural regions only (double-stranded with positive CROSS scores) can boost *CROSSalign* performances, while the use of unstructured regions only (single-stranded with negative CROSS scores) is prone to introduce noise. In general, *CROSSalign* could be improved by incorporating data relative to the global fold of the RNA in addition to the local properties predicted by *CROSS*. For example the information of NMR chemical shift could enhance the accuracy of secondary structure prediction (Zhang and Frank, 2018). Another important improvement would be to modify the approach to generate multiple profile alignments, which would allow a better identification of evolutionary traces associated with structural conservation. We are currently working on its implementation.

Our webserver is available at http://service.tartaglialab.com//new_submission/crossalign (documentation and tutorials are at the webpages http://service.tartaglialab.com/static_files/algorithms/crossalign/documentation.html and http://service.tartaglialab.com/static_files/algorithms/crossalign/tutorial.html) and allows to predict structural similarities between two (*Standard, OBE, Fragmented* modes) or more (*Dataset, Custom dataset*) RNA molecules. *CROSSalign* can be interrogated to search for structural similarities between thousands of lncRNA molecules and identifies regions with similar structures using a specific DTW algorithm (open begins and ends *OBE*).

As shown in the examples presented, *CROSSalign* is a versatile algorithm able to simplify the complex search for structural similarity among RNA molecules and shows great potential for the study of lncRNAs.

## Materials and methods

### Prediction of the RNA secondary structure: CROSS

Secondary structure profiles were generated using CROSS (Delli Ponti et al., 2017). CROSS models have been previously trained on data from high-throughput experiments [PARS: yeast and human transcriptomes (Kertesz et al., 2010; Wan et al., 2014) and icSHAPE: mouse transcriptome (Spitale et al., 2015)] as well as on low-throughput SHAPE (Watts et al., 2009) and high-quality NMR/X-ray data (Andronescu et al., 2008). The consensus model *Global Score* was trained and tested on independent sets of NMR/X-ray structures [11,670 training fragments, 5,475 testing fragments (Wu et al., 2015; Lorenz et al., 2016), see https://github.com/stanti/shapebenchmark/tree/master/benchmarkdata]. In the testing phase, single and double-stranded nucleotides were recognized with an AUC of 0.72 and a PPV of 0.74. Comparison with experimental SHAPE data shows similar performances (AUC of 0.76 and PPV of 0.76; see http://service.tartaglialab.com/static_files/algorithms/cross/documentation.html#5) and the details are reported in our previous publication (Delli Ponti et al., 2017).

In addition, as done with experimental SHAPE data, *Global Score* has been also used as a constraint in *RNAstructure* (Mathews et al., 1999; Reuter and Mathews, 2010). On the test set (Lorenz et al., 2016), *Global Score* was shown to increase the PPV of *RNAstructure* from 0.68 to 0.72, with remarkable improvements in 13 cases (from 0.44 to 0.72) and decreases the PPV in only three cases for which real SHAPE data does not improve performances. Moreover, using the partition function computed with *RNAstructure*, we previously calculated the AUC for each structure with and without CROSS constraints and observed an improvement from 0.81 to 0.86 when CROSS is integrated in the algorithm. We observed a similar trend using *RNAfold* (Lorenz et al., 2016) (the PPV increases from 0.67 to 0.70 using *Global Score* and the AUC remains at 0.85).

In the present study all the profiles were computed using the *Global Score* module: nucleotides with a score higher than 0 are predicted to be double-stranded and structured, while nucleotides with a score lower than 0 are single-stranded. Since the algorithm has no sequence length restriction and shows strong performances on both coding and non-coding RNAs (Delli Ponti et al., 2017) it was combined with DTW for pairwise comparison of structural profiles.

### Comparison of structural profiles: DTW

To compare two CROSS profiles, we used the *DTW* algorithm available in the R package *dtw* (Giorgino, 2009). The open begin and end (*OBE-DTW*) algorithm was employed to compare profiles of different lengths. Indeed, the standard *DTW* method imposes the same begins and ends to the two profiles that are compared, while *OBE-DTW* searches for the profile of shorter length within the other one. Accordingly, we used standard *DTW* to compare profiles of similar lengths (i.e., one sequence is < 3 times longer than the other), while *OBE-DTW* was employed to search for modules within larger profiles (e.g., RepA within the complete *XIST* sequence; XIST is ~45 times longer than RepA).

To generate pairwise structural comparisons, we used settings recommended in the *dtw* manual. The distance is computed with an asymmetric pattern and using the Manhattan distance, which is optimal for comparing profiles of different lengths. To avoid biases regarding the length of the profiles, the final *CROSSalign* distance is normalized for the lengths of both profiles using the internal function *normalizedDistance*. We also tested different normalizations of DTW outputs (including normalization by length of the shorter or longer profile) and we found that the normalization based on the lengths of both profiles is optimal. The function *index* was used to visualize the optimal path and to extract the matching coordinates between the two profiles.

### Statistical analysis

To compute the significance of a specific DTW score, we analyzed the statistical distributions generated using human lncRNAs of different lengths (200, 500, 1,000, 5,000 nucleotides). Hundred molecules for each class were employed to compute the distance between the classes. The distributions are set as a reference to compute the *p*-values in new analyses (Supplementary Table 4).

### Datasets

- lncRNA sequences were downloaded from ENSEMBL 82 using Biomart and specifying lincRNAs, for a total of 4,427 sequences for mouse and 8,176 for human (new releases are reported in the webpage).
- The complete viral genomes were downloaded from NCBI selecting ssRNA viruses having as host human or primates (for SIV), for a total of 292 complete genomes.
- The complete rRNA sequences were downloaded from NCBI.
- RepA, D2 and D4 were selected from the data publicly available from the work of Rivas et al. (2017). To keep consistency between the results we selected the same species between the two sets of multialignments, when available.
- The HIV strains were downloaded from HIV databases (https://www.hiv.lanl.gov/), selecting only complete genomes for a total of 4,804 sequences processable by CROSS.

### Sequence alignment

To compute the sequence alignments we used the browser version of EMBOSS-needleall, publicly available at http://www.bioinformatics.nl/cgi-bin/emboss/needleall. The tool was used with standard settings to speed up the calculation. The sequence identity was retrieved from the corresponding field from EMBOSS multiple output.

### Reverse engineering: from structure to sequence

To study how sequence similarity is related to structural similarity we created different sequences with the same secondary structure as RepA (*XIST*) and D2 (*HOTAIR*). To generate the reference structure we used *RNAfold* (Smola et al., 2016). We then generated different sequences encoding for the same previously generated structure. For this task we used the command line version *RNAinverse* from the Vienna suite (Lorenz et al., 2011). *RNAinverse* uses reverse folding engineering to generate several sequences whose minimum free energy matches the target structure. The tool was launched using standard parameters to generate 50 sequences for each structure.

### Comparisons with CMfinder

We compared the distances provided by *CROSSalign* with the multiple sequence alignment scores of *CMfinder* (Yao et al., 2006). For this analysis we selected 5 of the most complex datasets (i.e., highest number and longest sequences) from the test set of *CMfinder* (cobalamin, intron gp II, sbox, lysine, and histone 3). To compare the pairwise distances (*CROSSalign*) with the multiple alignment scores (*CMfinder*) we computed all the distances within the datasets and selected the lowest distance for each transcript. From low- (median) to high-confidence (top and bottom 5%) *CMfinder* scores, we observed an increase in the performances of *CROSSalign*, which indicates a good predictive power on the multiple alignment score.

### Comparisons with CMsearch

*CMsearch* is a method used to search a covariance model (CM) against a sequence database and provides a ranked list of the sequences with the most significant matches relative to the CM. Using the *CMbuild* package we built the CMs using as input the multiple alignments of the two individual reference sets (RepA and D2), obtained using *Clustal Omega*. The E-values of the CMs were obtained upon calibration with *CMcalibrate*. The calibrated CMs were then used to search for homologs in the positive and negative sets using the *CMsearch* approach. To build the AUC, we generated negative sets by shuffling the nucleotide composition of either RepA or D2. By running *RNAalifold* (Lorenz et al., 2011) to generate a consensus secondary structure on aligned sequences (Larkin et al., 2007), we did not obtain improvements for both RepA and D2 alignments.

### Webserver description

#### Input

The user should paste one or two RNA sequences in FASTA format into the dedicated form, providing an email address (optional) to receive a notification when the job is completed. The algorithm can be launched in 4 different modes, each of them being a specific variation of the DTW algorithm (Supplementary Figure 1).

- The *standard-DTW* is recommended for comparing structures of RNAs with similar lengths (i.e., one sequence is < 3 times longer than the other).
- *OBE-DTW* (open begins and ends) is a specific mode to search for a shorter profile within a longer one. This is the recommended mode when comparing profiles of very different sizes (i.e., one sequence is more than 3 times longer than the other). Please keep in mind that the sequence in the form of RNA input 1 will be searched for within RNA input 2, so the sequence in RNA input 1 should be shorter than the other.
- The *fragmented OBE-DTW* is a specific mode for searching for unknown secondary structure domains of one profile within the other. The secondary structure of RNA input 1 will be fragmented with a non-overlapping window of 200 nucleotides [optimal size to search secondary structure domains in large RNAs (Lange et al., 2012; Agostini et al., 2013)] Each fragment of RNA input 1 will then be searched for within the other sequence. This approach is the recommended mode when the user is not interested in the global similarity between two secondary structure profiles, but wants to search for an unknown domain conserved in both sequences. A minimum length of 600 nucleotides is recommended for fragmentation.
- The *dataset* mode allows the user to search for a single sequence within all the lincRNAs of a specific organism. Individual alignments are available for the top 20 pairs. In each case, the shorter profile is searched within the larger one following the *OBE-DTW* procedure. The organisms available are Human, Mouse, Rat, Macaque and Zebrafish. The lincRNAs were downloaded using Biomart (Ensemble 84). We also provide the latest release for the human lincRNAs (Ensemble 93).

#### Output

We report the *CROSSalign* score that measures the distance between two structures. The closer the score is to 0, the higher the similarity in terms of secondary structure. According to our statistical analysis, RNA molecules with a distance of 0.10 or higher are to be considered different in terms of secondary structure (see online *Documentation*).

The main image shows the overall structural similarity of the two profiles employed to calculate *CROSSalign* score (Supplementary Figure 2A). On the two axes the user will see the structural profiles obtained with CROSS for the two RNA sequences in input (score >0 means a double-stranded nucleotide; < 0 single-stranded). For a better visualization, the profiles are smoothed using a function previously defined (Delli Ponti et al., 2017).

The similarity is represented by the red path in the figure, obtained with the index function of the *dtw* package. The closer the path is to the diagonal, the more similar are the profiles. Vertical or horizontal paths are to be considered gaps, while diagonal paths highlight similar regions of the two profiles.

Since *OBE-DTW* allows the identification of the optimal starting/ending points of a match, the optimal match is reported in terms of coordinates relative to the larger profile (RNA input 2). The main plot shows the CROSS profiles of the optimal matching region selected by the *OBE-DTW* algorithm (Supplementary Figure 2B). In order to keep the gaps introduced by the *OBE-DTW* algorithm, the two profiles are not smoothed.

The *fragmented OBE-DTW* is a particular form of *DTW* optimized to search for all the possible structural domains of a particular sequence within another one. The main output is a scrolling table reporting the structural score, the beginning of the match, the end of the match and the *p*-value (Supplementary Figure 2C). All the values are computed with the procedure used for *OBE-DTW*. The table can also be downloaded as a .txt file. The same output is used for the *dataset* mode, but in this case the table can only be downloaded.

## Data availability statement

The code is publicly available under an open source license compliant with Open Source Initiative at *https://github.com/armaos/algorithm-crossalign*. The source code is deposited in a DOI-assigning repository *https://doi.org/10.5281/zenodo.1168294*.

## Author contributions

GT designed the work, RD implemented the approach with SM. AA and RD developed the server. GT, RD and SM wrote the paper. All authors reviewed the manuscript.

### Conflict of interest statement

The authors declare that the research was conducted in the absence of any commercial or financial relationships that could be construed as a potential conflict of interest.
