# Supplementary material for: A Method for RNA Structure Prediction Shows Evidence for Structure in lncRNAs
Source: Front Mol Biosci. 2018 Dec 3;5:111. doi: 10.3389/fmolb.2018.00111 (PMC6286970; doi:10.3389/fmolb.2018.00111)
Supplement: Supplementary file 5 [file Data_Sheet_1.PDF]

Supplementary Figures and Tables

Supplementary Figure 1. Main page of *CROSSalign* webserver. The user can upload fasta sequences and select a DTW mode for the task. The algorithm computes structural distances between two or more RNA sequences. Specifically, it 1) compares profiles of similar lengths (*standard-DTW*), 2) searches for domains of a short profile within a large one (*OBE-DTW*), 3) fragments a long sequence (*fragmented OBE-DTW*) or 4) searches for a profile within all the lncRNAs of a specific organism (*dataset*). See the **Tutorial** for more details.

Supplementary Figure 2. Outputs of *CROSSalign* webserver. (**A**) *Standard-DTW*. On the two axes there are the secondary structure profiles obtained with CROSS, while in the main plot it is reported the optimal path (highlights the regions of similarity). (**B**) *OBE-DTW* overlap of the best matching region between the two secondary structure profiles (obtained with CROSS). (**C**) *Fragmented OBE-DTW* output table showing structural distance, starting and ending positions of the match and the p-values for all the fragments of the input profile. See the **Tutorial** for more details.

Supplementary Figure 3. (**A**) Structural distances correlation between *RNAstructure* and crystallographic profiles (22x22 structures). (**B**) Structural distances correlation between *RNAfold* (Vienna suite) and crystallographic profiles (22x22 structures).

Supplementary Figure 4. (**A**) Correlation between structural distances (*RNAstructure*) and sequence similarity. In this case the clusters previously identified are disrupted. (**B**) Correlation between structural distances (*RNAfold*) and sequence similarity. In this case the clusters previously identified are disrupted.

Supplementary Figure 5. (**A**) Global performances of CROSSalign on the test set cases of CMfinder. From low- (median) to high-confidence (top and bottom 5%) of CMfinder scores, the performances of our tool increase, which indicates good predictive power on the multiple alignment score (AUC 0.67, 0.70, 0.75 and 0.79). (**B**) Barplot showing the performances (AUC) of *CROSSalign* for the larges dataset (Cobalamin). From low- (median) to high-confidence (top and bottom 5%) of the CMfinder score distribution the AUC raises up to 0.98.

Supplementary Figure 6. Distributions of the sequence identities of the reversed engineered sequences compared with the original RepA (red) and D2 (green).

Supplementary Figure 7. (**A**) Structural difference with respect to human (structural distance *100) for *XIST* RepA in 10 different species. The primates cluster together. (**B**) Sequence distances from human calculated as (100-sequence similarity)% for 10 different species. The primates' cluster cannot be identified by primary sequence.

Supplementary Figure 8. Dendrogram showing the species tree for *XIST* RepA obtained using structural distance.

Supplementary Figure 9. (**A**) *CROSS* performances on *HOTAIR* SHAPE data. From low- (median) to high-confidence (top and bottom 5%) of the SHAPE distribution, we observed an increase in the performances (AUC 0.73, 0.84, 0.86, 0.89). (**B**) ROC curve on the top and bottom 25% of the SHAPE distribution for HOTAIR (AUC 0.84).

Supplementary Figure 10. ROC curve of *CMsearch* for reverse engineered sequences with the same structure as D2.

Supplementary Figure 11. (**A**) Structural differences with respect to human (structural distance *100) for *HOTAIR* D2 of 10 different species. The primates tend to cluster together. (**B**) Sequence difference with respect to human [(100-sequence similarity)%] for 10 different species. The primates' cluster can also be identified by primary sequence.

Supplementary Figure 12. (**A**) Differences in structure from human (structural distance *100) for the D4 of 10 different species. The primates tend to cluster together. (**B**) Sequence distances from human calculated as (100-sequence similarity)% for 10 different species. The primates' cluster can be identified by primary sequence.

Supplementary Table 1. RepA: Reference, positive and negative sequences D2: Reference, positive and negative sequences.

Supplementary Table 2. Table summarizing the results for the lncRNAs reported in our work.

Supplementary Table 3. Table reporting the 3 best candidates for each HIV domain. The results discussed in the main text are reported in bold.

Supplementary Table 4. (**A**) Means and standard deviations for structural distances of reference distributions. The *standard-DTW* was used to compare profiles of similar length, while *OBE-DTW* for the other cases. (**B**) P-values at 1% of the structural distances. The *standard-DTW* was used for profiles of similar length, while *OBE-DTW* for the all others.
