# Supplementary material for: A Method for RNA Structure Prediction Shows Evidence for Structure in lncRNAs
Source: Front Mol Biosci. 2018 Dec 3;5:111. doi: 10.3389/fmolb.2018.00111 (PMC6286970; doi:10.3389/fmolb.2018.00111)

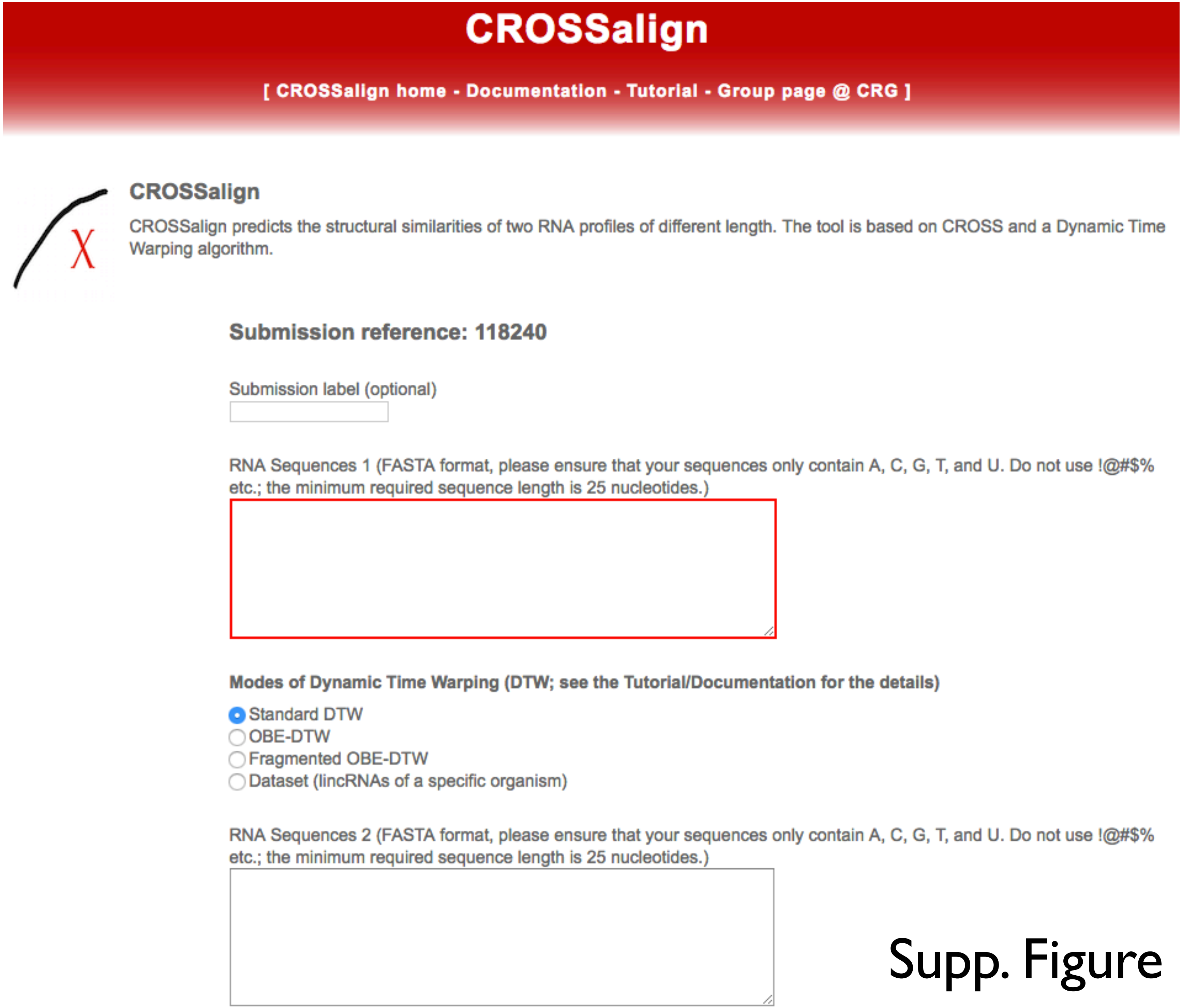

Supp. Figure 1

Supp. Figure 2

A

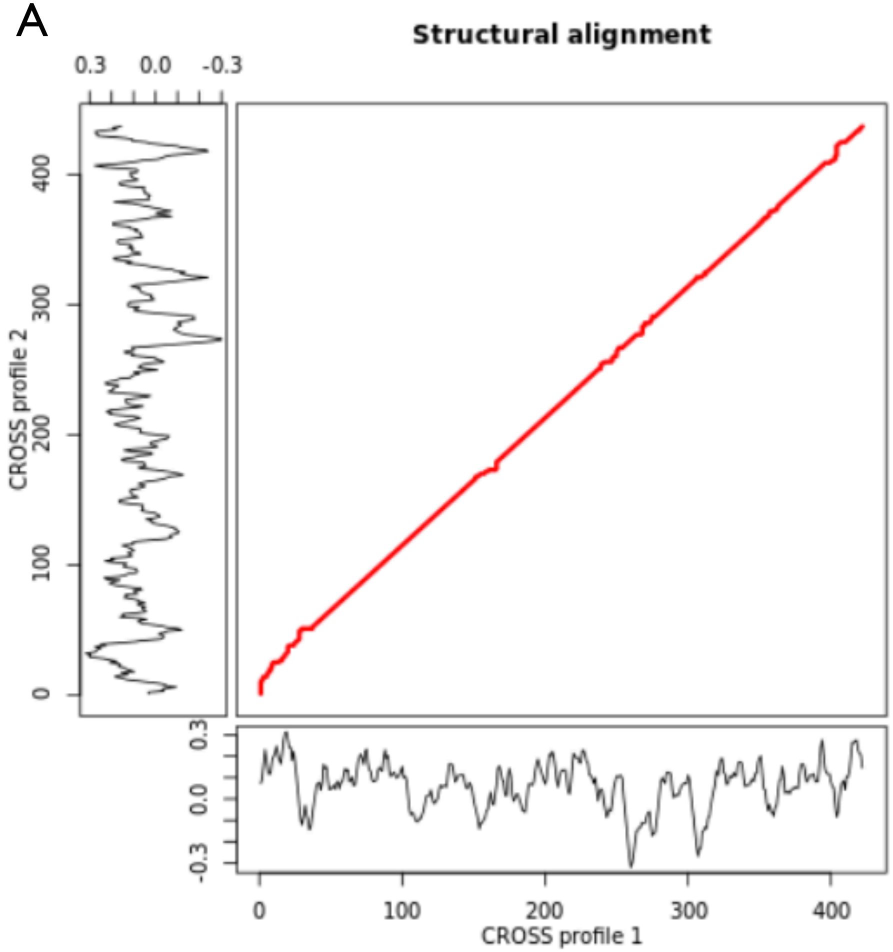

B

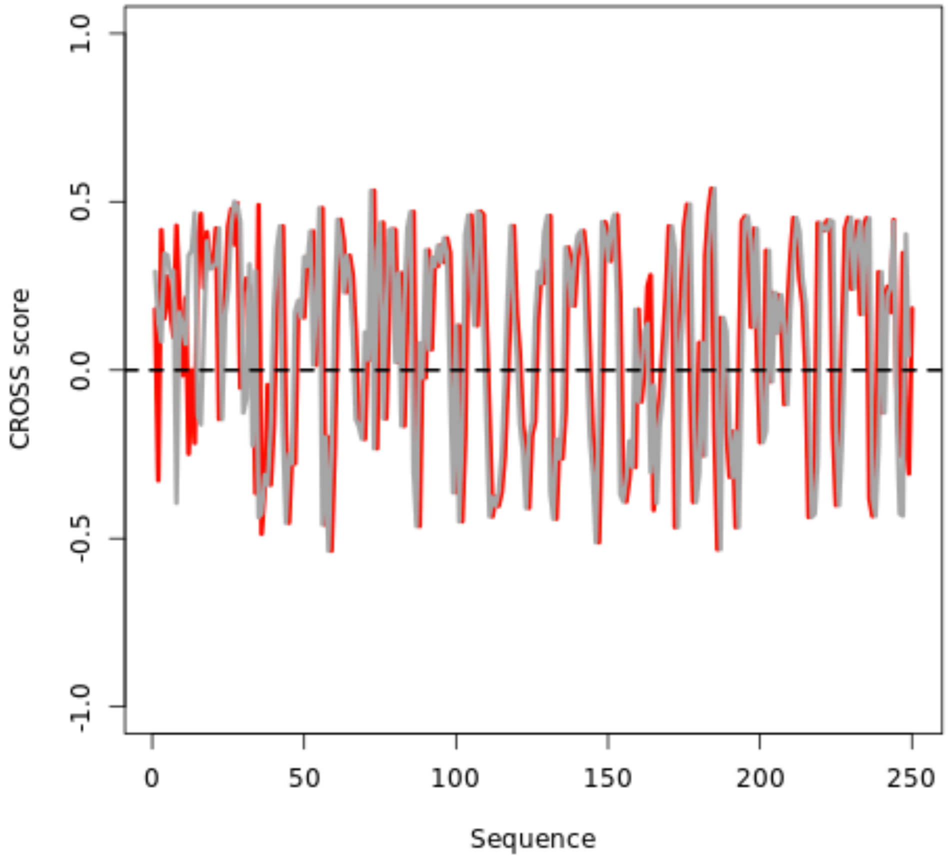

C

Download the complete set of data

HIV1_5600	0.07972	5397	5597	<1.0e-06
HIV1_5800	0.089865	5619	5819	<1.0e-06
HIV1_6000	0.08903	3450	3650	<1.0e-06
HIV1_6200	0.086285	8021	8221	<1.0e-06
HIV1_6400	0.095765	6193	6393	7.5e-03
HIV1_6600	0.070175	6389	6589	<1.0e-06
HIV1_6800	0.090475	5989	6189	<1.0e-06
HIV1_7000	0.09189	8055	8255	<1.0e-06
HIV1_7200	0.08855	3950	4150	<1.0e-06
HIV1_7400	0.09012	8777	8977	<1.0e-06
HIV1_7600	0.09175	6612	6812	<1.0e-06
HIV1_7800	0.0697	7555	7755	<1.0e-06
HIV1_8000	0.06192	7771	7971	<1.0e-06
HIV1_8200	0.08324	7971	8171	<1.0e-06

Supp. Figure 3

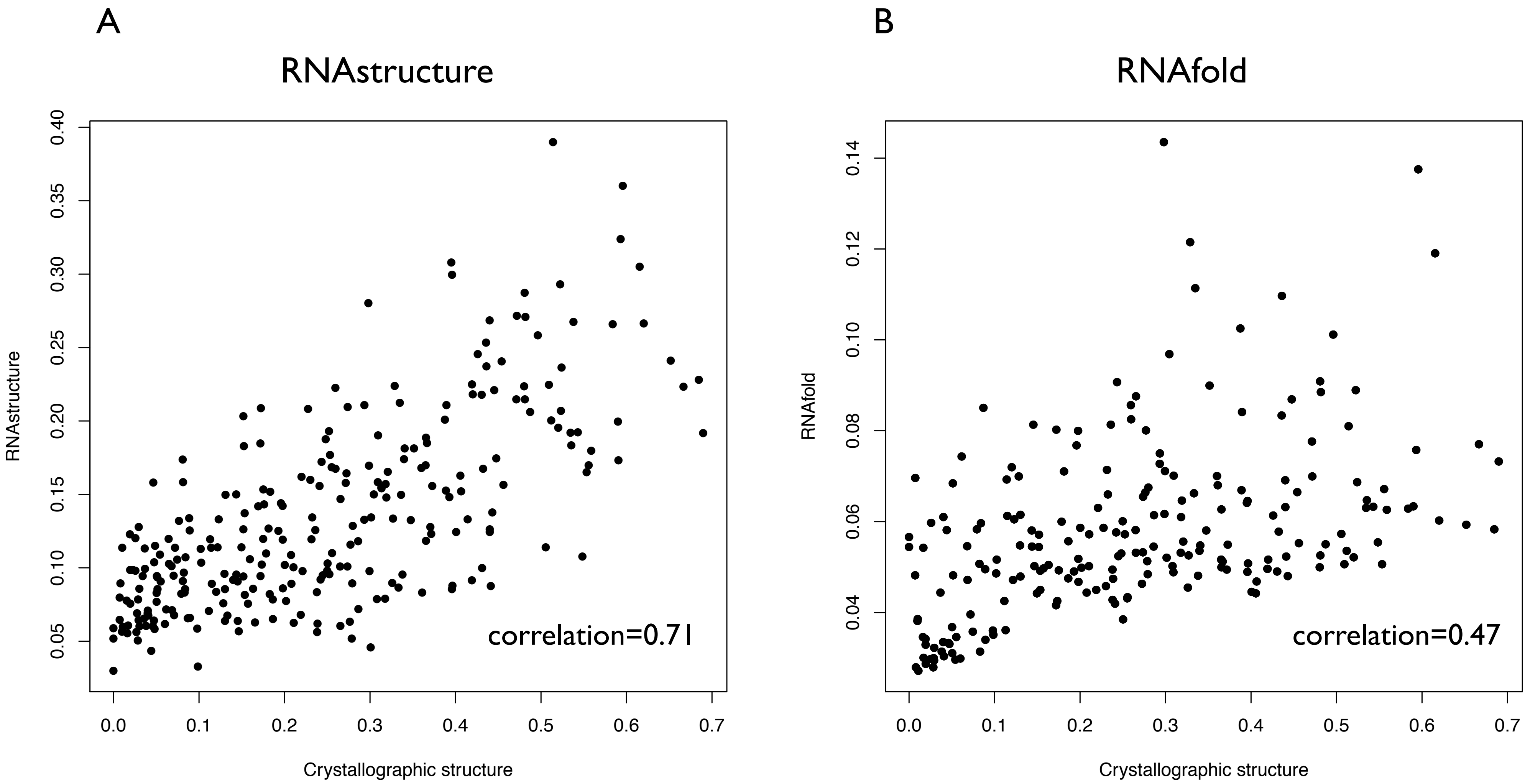

Supp. Figure 4

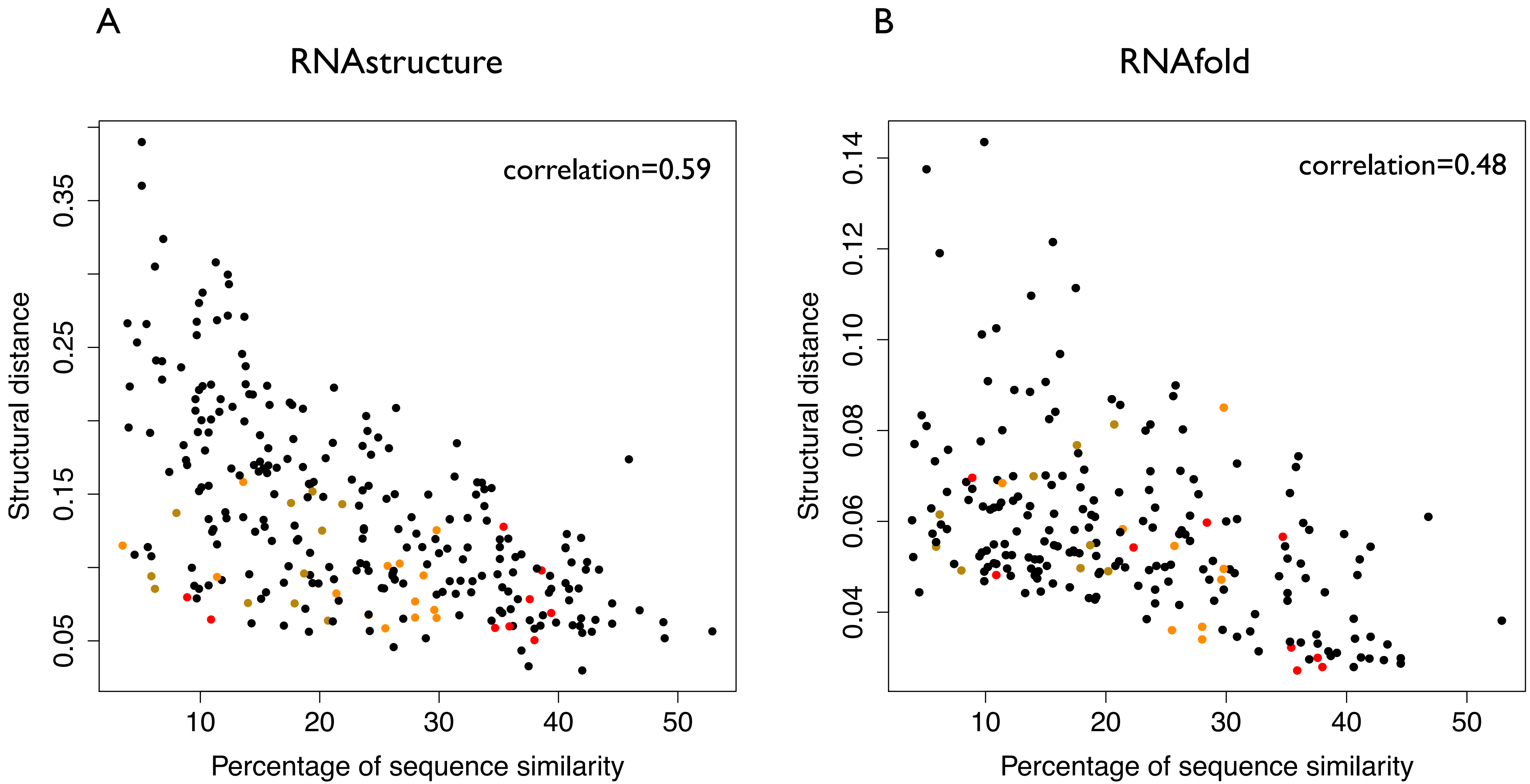

Supp. Figure 5

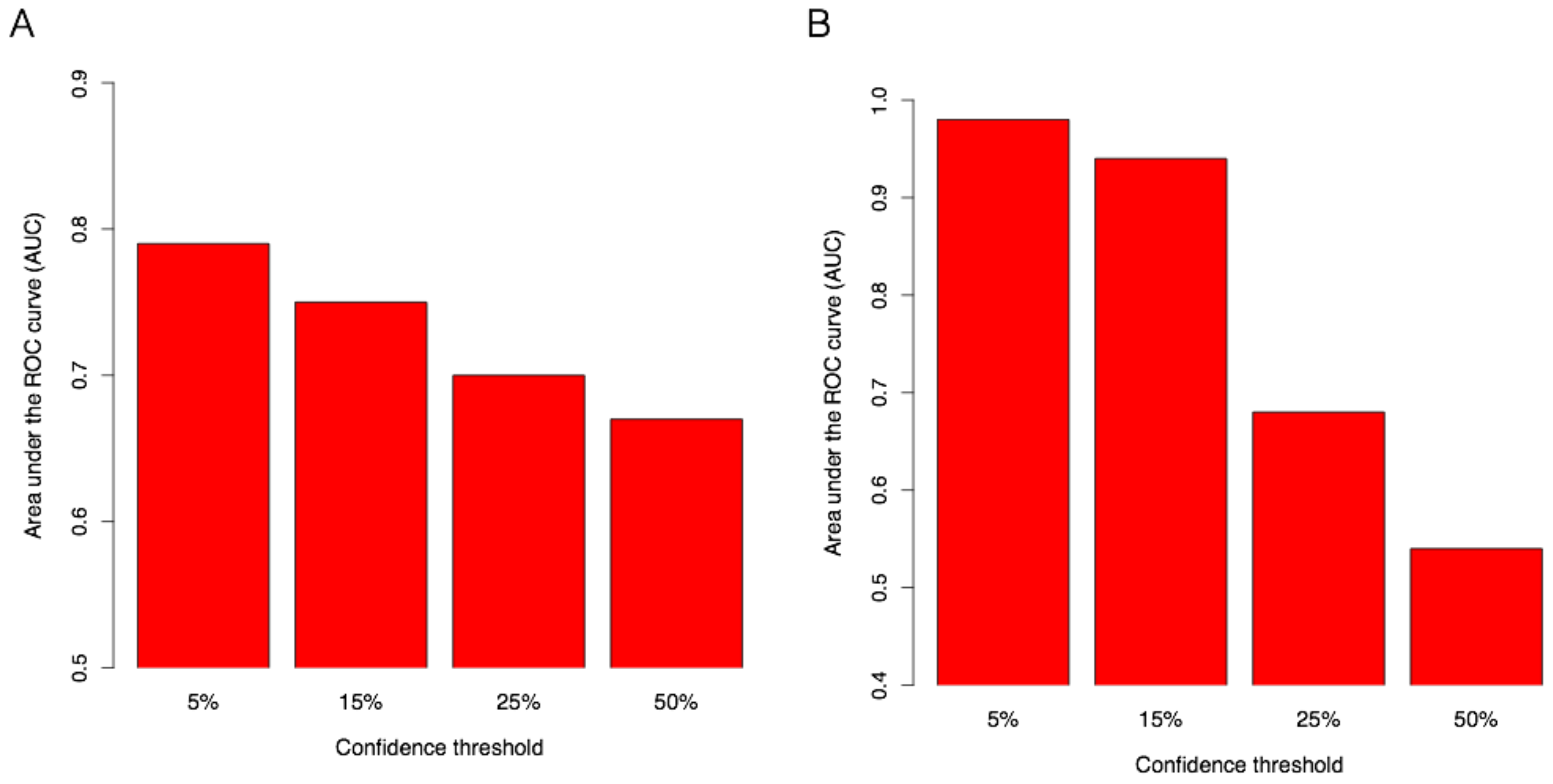

Supp. Figure 6

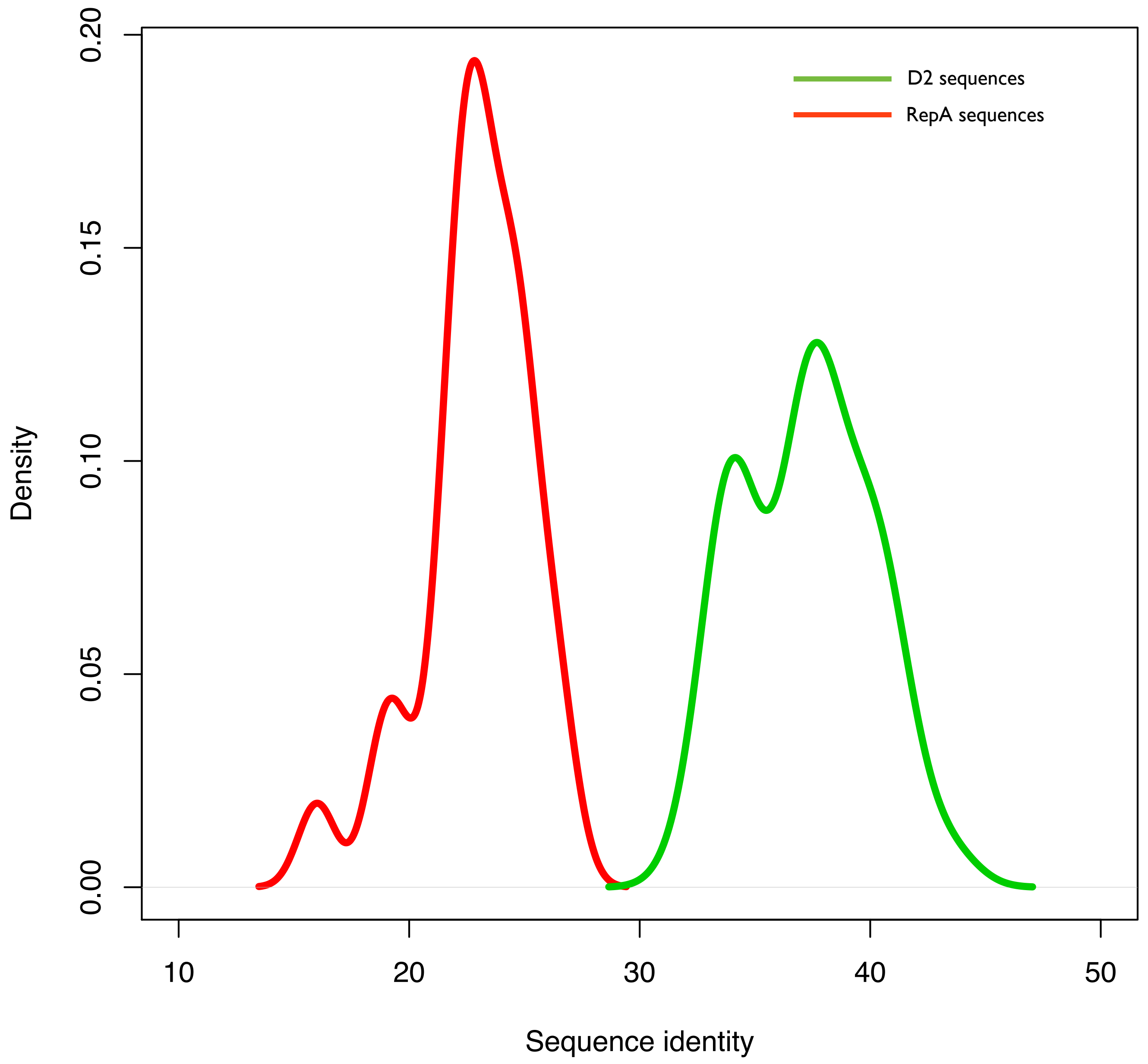

Supp. Figure 7

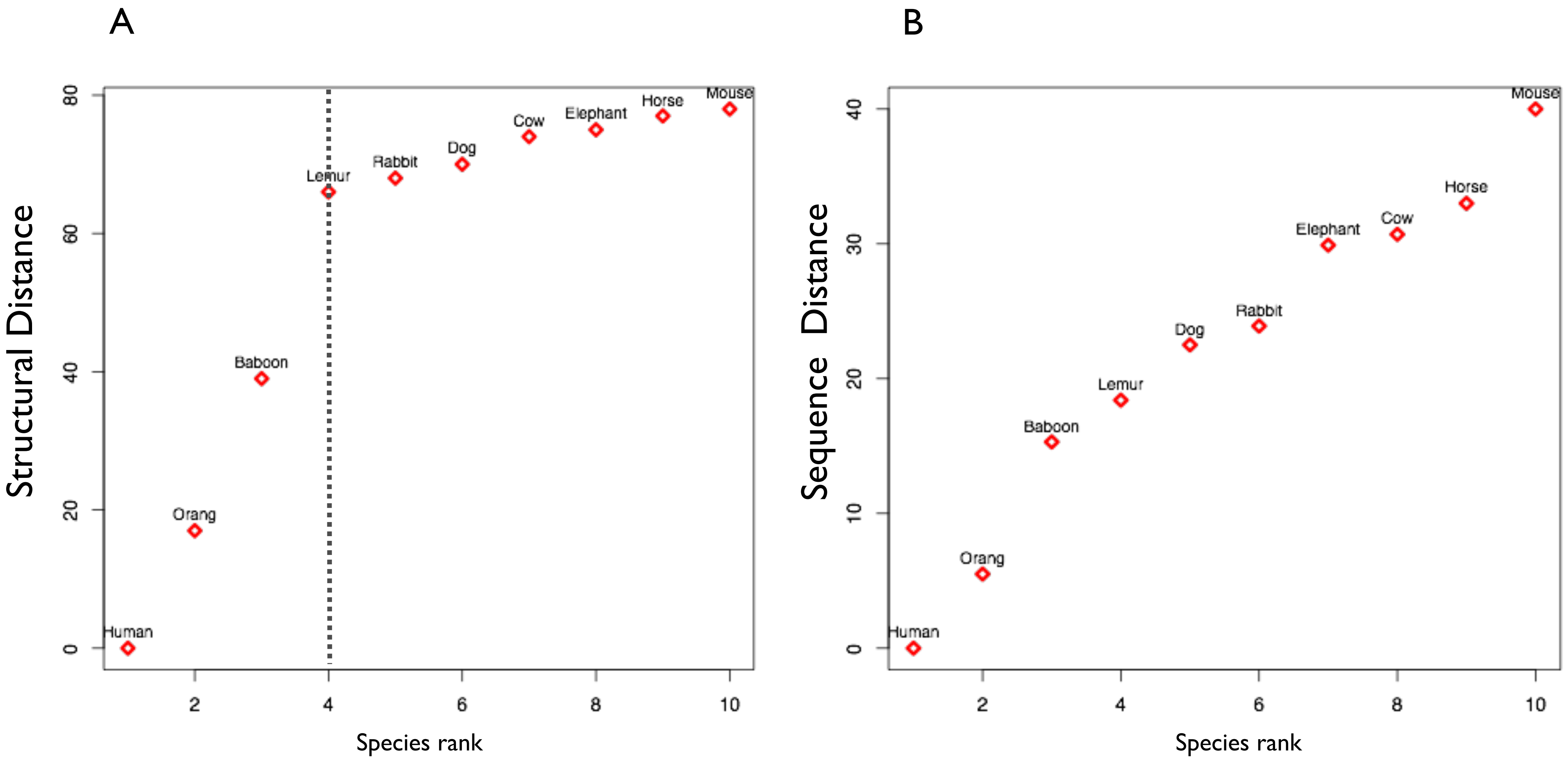

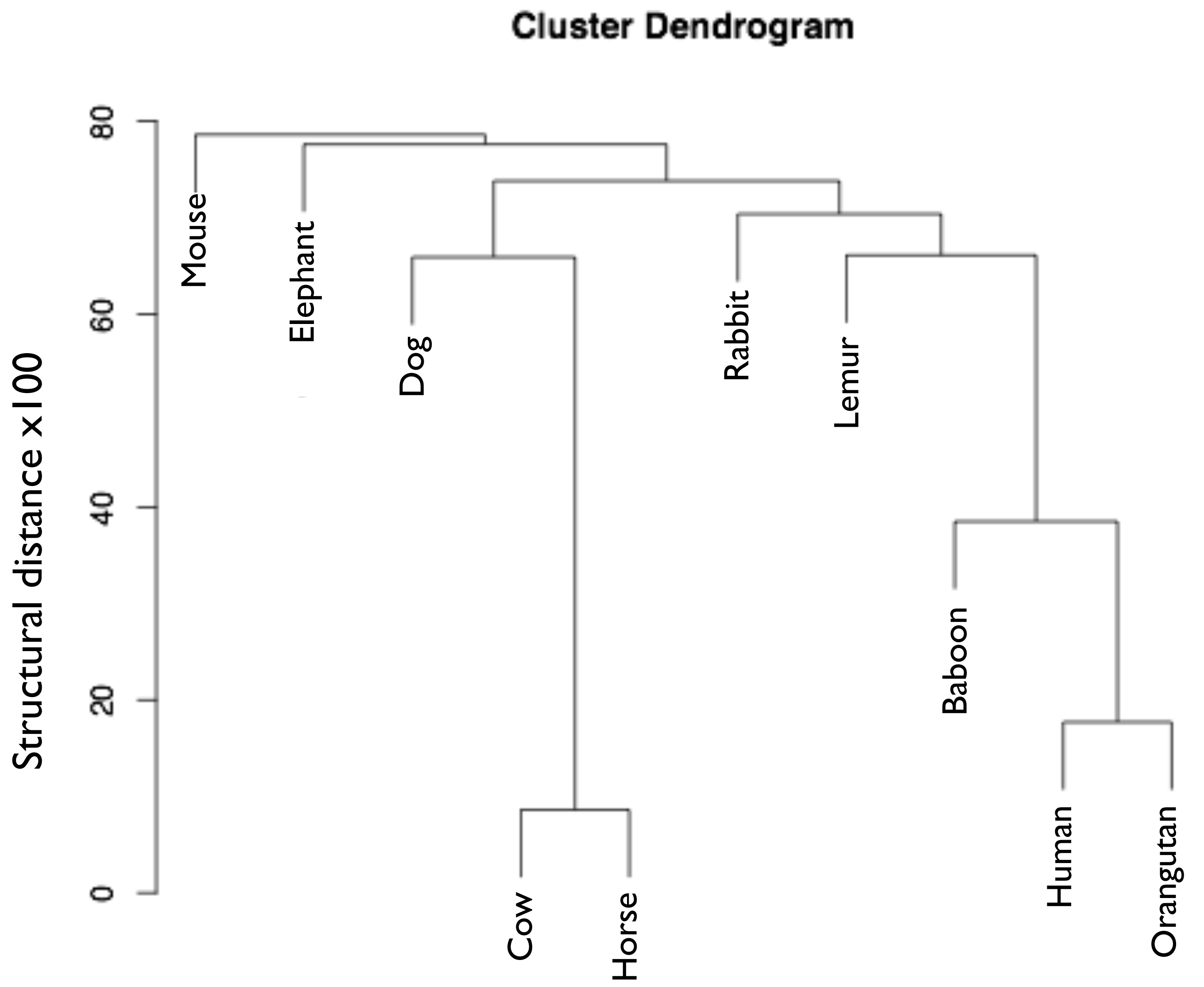

Supp. Figure 8

Supp. Figure 9

A

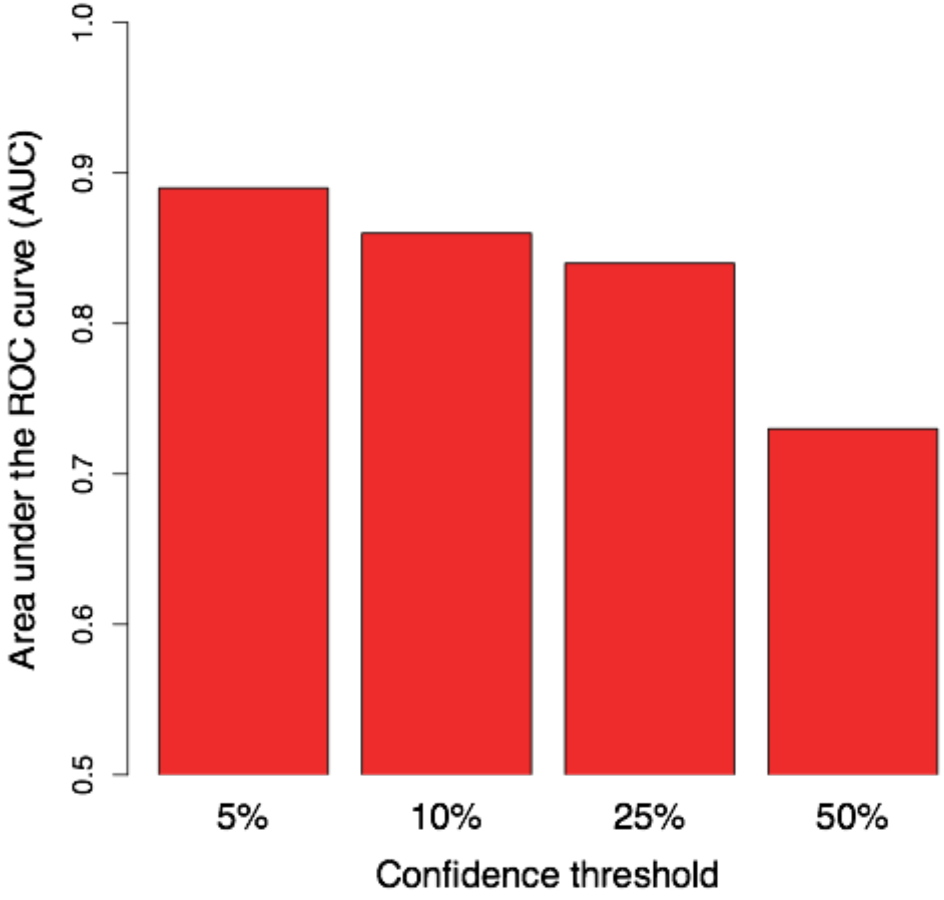

B

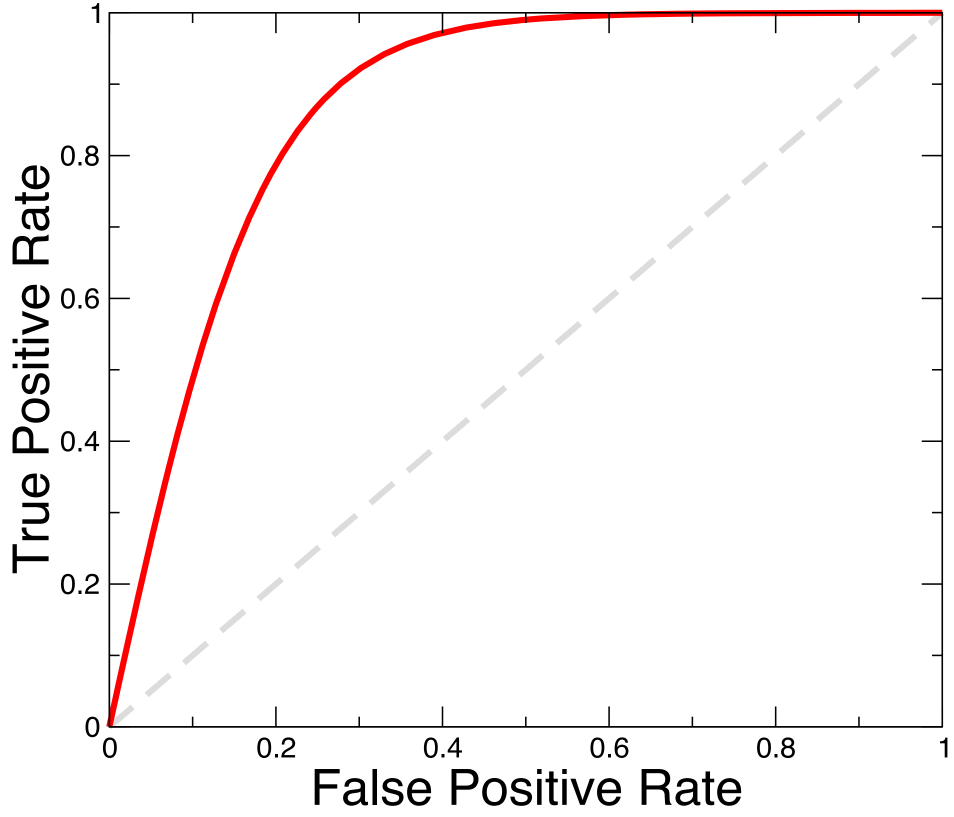

Supp. Figure 10

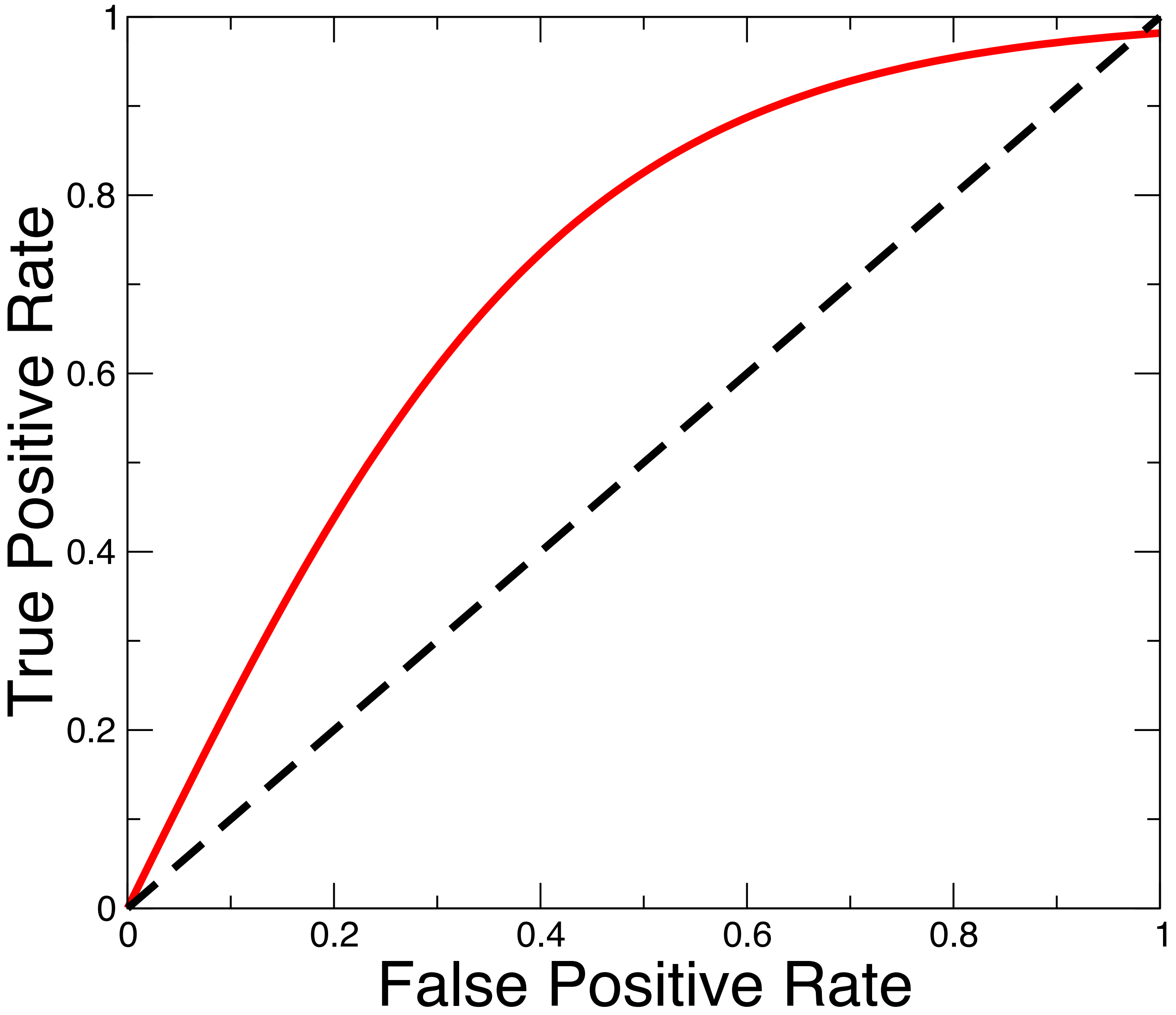

Supp. Figure 11

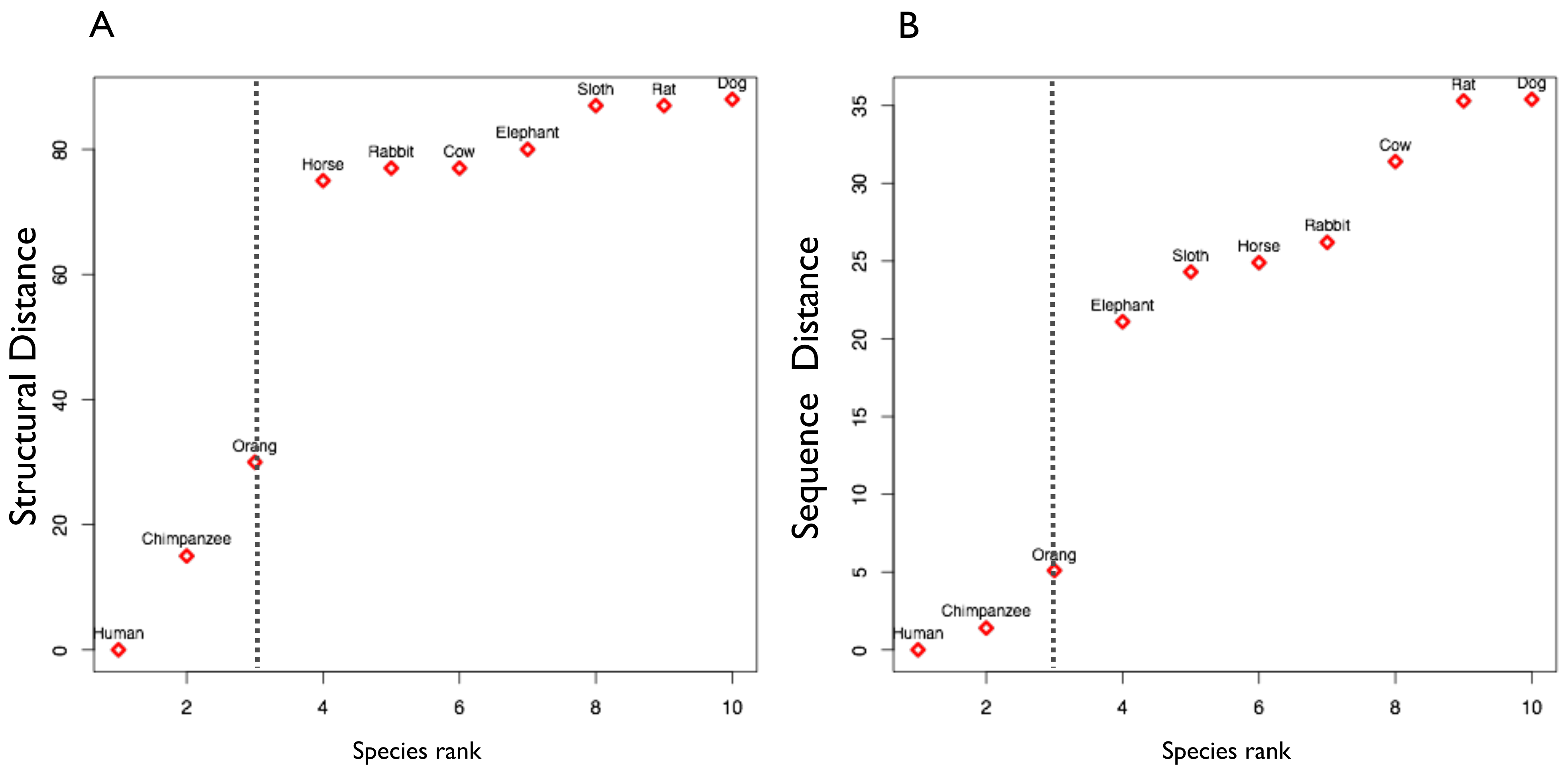

Supp. Figure 12

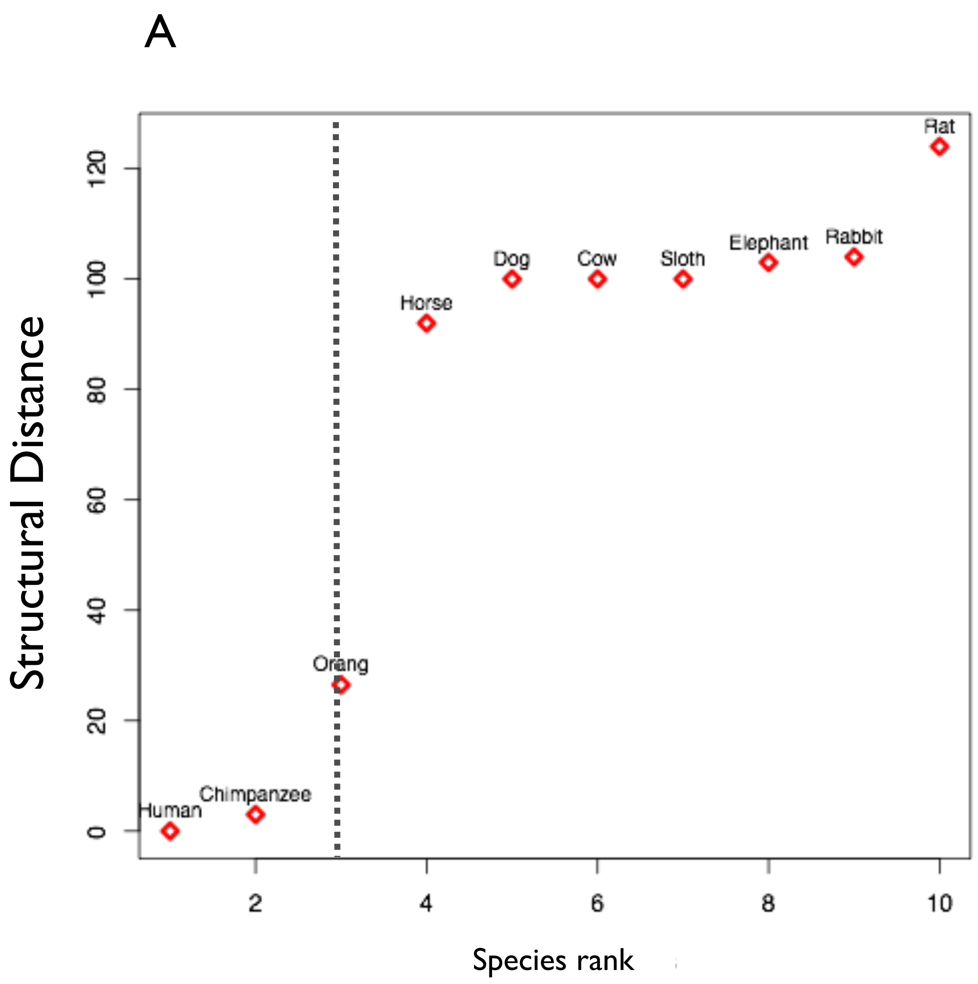

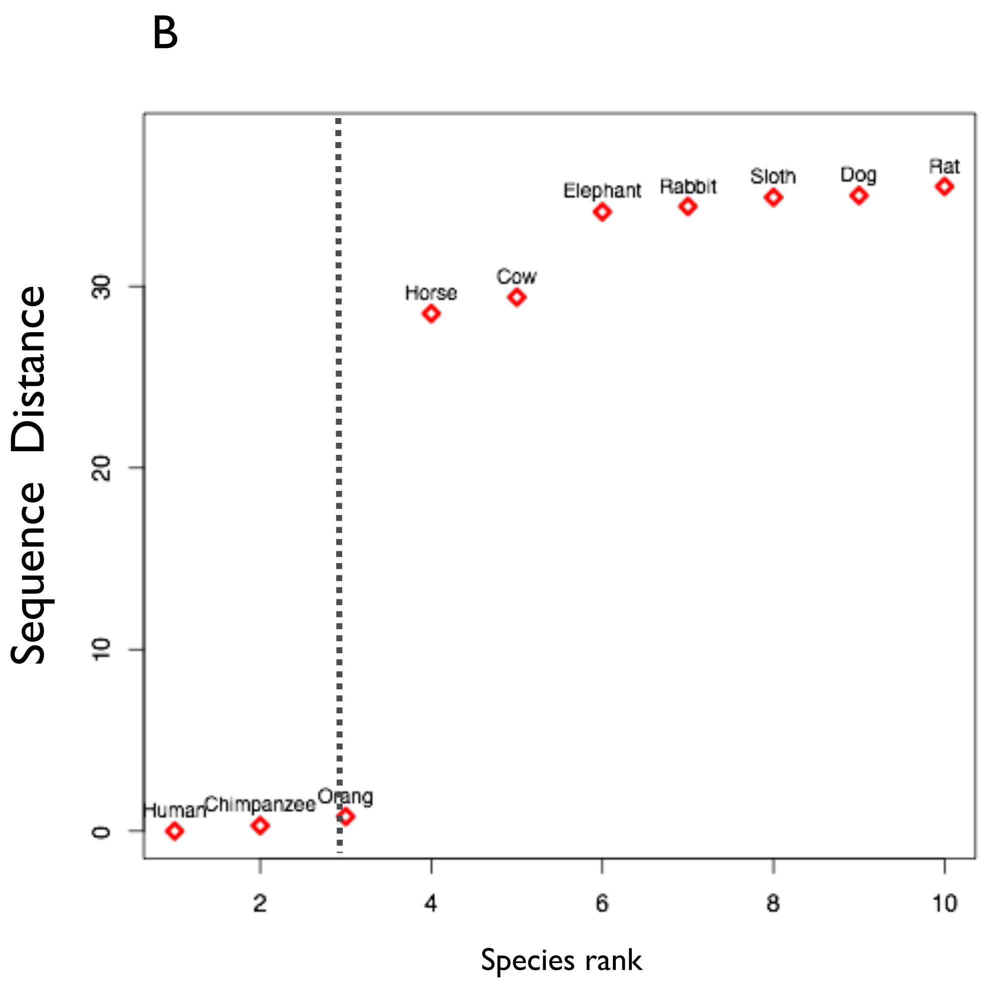

Supplement: Supplementary file 6 [file Data_Sheet_2.PDF]
